# Modeling antisense oligonucleotide therapy in *MECP2* duplication syndrome human iPSC-derived neurons reveals gene expression programs responsive to MeCP2 levels

**DOI:** 10.1093/hmg/ddae135

**Published:** 2024-09-15

**Authors:** Sameer S Bajikar, Yehezkel Sztainberg, Alexander J Trostle, Harini P Tirumala, Ying-Wooi Wan, Caroline L Harrop, Jesse D Bengtsson, Claudia M B Carvalho, Davut Pehlivan, Bernhard Suter, Jeffrey L Neul, Zhandong Liu, Paymaan Jafar-Nejad, Frank Rigo, Huda Y Zoghbi

**Affiliations:** Department of Molecular and Human Genetics, Baylor College of Medicine, One Baylor Plaza, Houston, TX 77030, United States; Jan and Dan Duncan Neurological Research Institute at Texas Children’s Hospital, 1250 Moursund Street, Houston, TX 77030, United States; Department of Cell Biology, University of Virginia, 1340 Jefferson Park Avenue, Charlottesville, VA 22903, United States; Department of Biomedical Engineering, University of Virginia, 415 Lane Road, Charlottesville, VA 22903, United States; Department of Molecular and Human Genetics, Baylor College of Medicine, One Baylor Plaza, Houston, TX 77030, United States; Jan and Dan Duncan Neurological Research Institute at Texas Children’s Hospital, 1250 Moursund Street, Houston, TX 77030, United States; Jan and Dan Duncan Neurological Research Institute at Texas Children’s Hospital, 1250 Moursund Street, Houston, TX 77030, United States; Department of Pediatrics, Baylor College of Medicine, One Baylor Plaza, Houston, TX 77030, United States; Department of Molecular and Human Genetics, Baylor College of Medicine, One Baylor Plaza, Houston, TX 77030, United States; Jan and Dan Duncan Neurological Research Institute at Texas Children’s Hospital, 1250 Moursund Street, Houston, TX 77030, United States; Department of Molecular and Human Genetics, Baylor College of Medicine, One Baylor Plaza, Houston, TX 77030, United States; Jan and Dan Duncan Neurological Research Institute at Texas Children’s Hospital, 1250 Moursund Street, Houston, TX 77030, United States; Department of Cell Biology, University of Virginia, 1340 Jefferson Park Avenue, Charlottesville, VA 22903, United States; Pacific Northwest Research Institute, 720 Broadway, Seattle, WA 98122, United States; Pacific Northwest Research Institute, 720 Broadway, Seattle, WA 98122, United States; Jan and Dan Duncan Neurological Research Institute at Texas Children’s Hospital, 1250 Moursund Street, Houston, TX 77030, United States; Department of Pediatrics, Baylor College of Medicine, One Baylor Plaza, Houston, TX 77030, United States; Section of Neurology and Developmental Neuroscience, Department of Pediatrics, Baylor College of Medicine, One Baylor Plaza, Houston, TX 77030, United States; Texas Children’s Hospital, 6621 Fannin Street, Houston, TX 77030, United States; Department of Pediatrics, Baylor College of Medicine, One Baylor Plaza, Houston, TX 77030, United States; Section of Neurology and Developmental Neuroscience, Department of Pediatrics, Baylor College of Medicine, One Baylor Plaza, Houston, TX 77030, United States; Texas Children’s Hospital, 6621 Fannin Street, Houston, TX 77030, United States; Vanderbilt Kennedy Center, 110 Magnolia Circle, Vanderbilt University Medical Center, Nashville, TN 37232, United States; Jan and Dan Duncan Neurological Research Institute at Texas Children’s Hospital, 1250 Moursund Street, Houston, TX 77030, United States; Department of Pediatrics, Baylor College of Medicine, One Baylor Plaza, Houston, TX 77030, United States; Ionis Pharmaceuticals, 2855 Gazelle Court, Carlsbad, CA 92010, United States; Ionis Pharmaceuticals, 2855 Gazelle Court, Carlsbad, CA 92010, United States; Department of Molecular and Human Genetics, Baylor College of Medicine, One Baylor Plaza, Houston, TX 77030, United States; Jan and Dan Duncan Neurological Research Institute at Texas Children’s Hospital, 1250 Moursund Street, Houston, TX 77030, United States; Department of Pediatrics, Baylor College of Medicine, One Baylor Plaza, Houston, TX 77030, United States; Section of Neurology and Developmental Neuroscience, Department of Pediatrics, Baylor College of Medicine, One Baylor Plaza, Houston, TX 77030, United States; Texas Children’s Hospital, 6621 Fannin Street, Houston, TX 77030, United States; Howard Hughes Medical Institute, Baylor College of Medicine, Houston, TX 77030, United States

**Keywords:** genetic syndromes, copy-number gain, iPSC derived neurons, transcriptomics, genomic disorder

## Abstract

Genomic copy-number variations (CNVs) that can cause neurodevelopmental disorders often encompass many genes, which complicates our understanding of how individual genes within a CNV contribute to pathology. *MECP2* duplication syndrome (MDS or MRXSL in OMIM; OMIM#300260) is one such CNV disorder caused by duplications spanning methyl CpG-binding protein 2 (*MECP2*) and other genes on Xq28. Using an antisense oligonucleotide (ASO) to normalize *MECP2* dosage is sufficient to rescue abnormal neurological phenotypes in mouse models overexpressing *MECP2* alone, implicating the importance of increased *MECP2* dosage within CNVs of Xq28. However, because MDS CNVs span *MECP2* and additional genes, we generated human neurons from multiple MDS patient-derived induced pluripotent cells (iPSCs) to evaluate the benefit of using an ASO against *MECP2* in a MDS human neuronal context. Importantly, we identified a signature of genes that is partially and qualitatively modulated upon ASO treatment, pinpointed genes sensitive to MeCP2 function, and altered in a model of Rett syndrome, a neurological disorder caused by loss of MeCP2 function. Furthermore, the signature contained genes that are aberrantly altered in unaffected control human neurons upon MeCP2 depletion, revealing gene expression programs qualitatively sensitive to MeCP2 levels in human neurons. Lastly, ASO treatment led to a partial rescue of abnormal neuronal morphology in MDS neurons. All together, these data demonstrate that ASOs targeting *MECP2* benefit human MDS neurons. Moreover, our study establishes a paradigm by which to evaluate the contribution of individual genes within a CNV to pathogenesis and to assess their potential as a therapeutic target.

## Introduction

Copy-number variations (CNVs), such as genomic duplications and deletions leading to dysregulation of dosage-sensitive genes are known causes of neurodevelopmental disorders like autism spectrum disorders and intellectual disability [1–3]. Studying these CNV-disorders, i.e. genomic disorders, can be difficult as the CNVs span multiple genes, making the contribution of each individual gene within the CNV to the clinical phenotypes difficult to assess. Thus, it is especially challenging to determine the effect of therapeutically targeting a single gene within a CNV containing many genes. 

*MECP2* duplication syndrome (MDS or referred to as MRXSL in OMIM; OMIM#300260) is an exemplar CNV neurodevelopmental disorder caused by genomic duplications spanning methyl CpG binding protein 2 (*MECP2*) [4–6]. MDS is a rare disorder that primarily affects males, with an estimated incidence of 0.12/100000 person-years in males [7]. MDS is characterized by infantile hypotonia, progressive motor difficulties, intellectual disability, epilepsy, recurrent respiratory infections, and premature death [6, 8–10]. Although CNVs that cause MDS can include multiple genes along Xq28, the minimal overlapped region includes only *MECP2* and interleukin-1 receptor-associated kinase 1 (*IRAK1*) [11]. Furthermore, overexpression of human *MECP2* in mice causes neurological dysfunction, establishing that a duplication of *MECP2* alone is pathogenic [12].

We recently demonstrated that the neurological abnormalities in mouse models of MDS can be reversed by reducing MeCP2 protein levels in the brain using an antisense oligonucleotide (ASO) targeting *MECP2* [13, 14]. ASOs are small oligonucleotides comprised of chemically modified nucleic acids that specifically hybridize with the target transcript. When hybridized, the DNA–RNA heteroduplex is recognized and cleaved by the endogenous nuclease, ribonuclease (RNase) H1, resulting in down-regulation of the target transcript [15]. These preclinical studies, coupled with the success of ASO treatments for other neurological disorders [16–18], indicate that ASOs could be used to treat MDS in humans by reducing aberrantly high MeCP2 protein levels.

*MECP2* encodes a methyl-cytosine binding protein that regulates gene expression in neurons [19–21]. Proper *MECP2* dosage is critical for normal brain function, as loss-of-function mutations in *MECP2* cause Rett syndrome (RTT; OMIM#312750) [22]. RTT affects ~1 in 10 000 females and is characterized by a period of normal development followed by regression and neurological dysfunction including stereotypies, breathing difficulties, loss of language, intellectual disability, and seizures [23–26]. RTT has been modeled in mice through germline mutations in *Mecp2* [27, 28]. Molecular characterization of RTT mouse models revealed that loss of *Mecp2* causes the dysregulation of hundreds to thousands of genes. Comparing these dysregulated genes in RTT to those dysregulated in mouse models of MDS identified a common subset of transcripts dysregulated in an opposing manner, demonstrating that the transcriptome is responsive to MeCP2 levels [20, 29–31]. RTT has also been modeled by postnatally deleting *Mecp2*, which demonstrated that neuronal function is acutely sensitive to MeCP2 function [32–34]. When MeCP2 levels are acutely decreased genetically or pharmacologically in mouse models of MDS, gene expression rescue precedes behavioral rescue, suggesting that normalizing gene expression downstream of MeCP2 is important to restoring normal neuronal function in MDS [13, 14]. Therefore, the transcriptome and molecular readouts are an ideal outcome to assess therapeutic benefit for *MECP2*-related disorders, and genes that are modulated by acute changes in MeCP2 levels may help us discover expression programs that are either involved in driving pathogenesis when MeCP2 is depleted in healthy neurons or responsive in reversal of pathogenesis when MeCP2 levels are normalized in MDS neurons.

With these considerations, we sought to establish a human neuronal system of MDS to assess the molecular benefit of reducing *MECP2* dosage alone. Patient-derived cells can be reprogrammed into induced pluripotent stem cells (iPSCs) that can then be differentiated into the disease-relevant cell type, which allows the generation of *in vitro* neuronal avatars of disease to understand disease mechanism and test potential therapeutics [35, 36]. iPSCs and iPSC-derived neurons have been used to study *MECP2* loss-of-function and RTT and to study MDS, though the effects of altered MeCP2 dosage on the human transcriptome have not been systematically studied [37–40].

In this study, we generated human neurons from iPSCs derived from unaffected control and MDS individuals with varying CNVs spanning *MECP2*. With these patient-derived neurons, we utilized transcriptomics to identify a gene expression signature of *MECP2* duplication. Like observations in MDS mouse models, human MDS neurons exhibit global transcriptional dysregulation. Next, we discovered that ASO treatment leads to a partial and qualitative rescue of gene expression. To uncover gene expression programs sensitive to MeCP2 levels, we compared the MDS neuronal disease signature first to RTT patient iPSC-derived neurons and found a program of genes inversely dysregulated between RTT and MDS neurons, mirroring observations made in mouse models. Second, acute depletion of MeCP2 from unaffected control neurons using ASO treatment identified a program of genes acutely responsive to MeCP2 levels. Importantly, both these gene expression programs were partially and qualitatively modulated in MDS neurons treated with ASO, contributing to the overall gene expression modulation seen in MDS neurons after ASO treatment. Lastly, treating MDS neurons for two weeks with ASO led to a mild improvement in neuronal morphology. These results enumerate the benefits of restoring *MECP2* expression in MDS human neurons using ASO therapy. More broadly, our study establishes a paradigm of how to dissect the contribution of single genes within other CNV/duplication disorders.

## Results

### Generation of induced pluripotent cells from *MECP2* duplication patients

**Figure 1:**
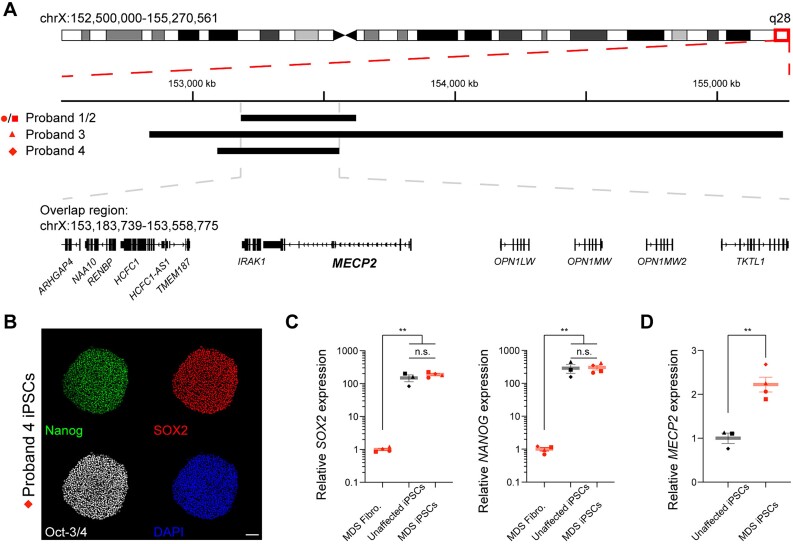
Generation and characterization of patient-derived *MECP2* duplication syndrome induced pluripotent cells (iPSCs). (A) Schematic of genomic duplications on the X-chromosome for probands in this study. The line denotes the size for each proband. Proband 1 and 2 are siblings that have identical duplications. Genes located within the overlapped region of all rearrangements of chrXq28 are below. Coordinates and gene annotations are based on hg19 genome assembly. Shapes correspond to each proband used throughout the study. (B) Validation of pluripotency markers (Nanog, SOX2, Oct-3/4) in MDS patient-derived iPSCs. Scale bar = 100 μm. (C and D) Gene expression quantification of pluripotency markers (*SOX2*, *NANOG*) in iPSCs compared to the parental MDS fibroblasts (C) and *MECP2* expression in MDS iPSCs compared to unaffected control iPSC lines (D) by qPCR. Graphs display mean ± sem of *n* = 3-4 biological replicates per genotype, individual patients are depicted with a separate shape. Differences in gene expression were assessed by Student’s t-test by genotype (^*^^*^P < 0.01).

We collected and cultured fibroblasts from four probands clinically and genetically diagnosed with MDS. The precise genomic coordinates for the beginning and end of the duplication of Xq28 were mapped using customized array comparative genomic hybridization (aCGH). The genomic coordinates for Probands 1,2, and 4 were mapped in a previous study [41], while the genomic coordinates for Proband 3 are reported in this study. Two probands are siblings and share identical duplications of chrX:153 183 739–153 623 000 (Probands 1 and 2) (GRCh37/hg19). Proband 3 has a duplication of chrX:152 834 323–155 251 054, while Proband 4 has a duplication of chrX:153 093 425–153 558 775 (Fig. 1A, Supplementary Material, Fig. S1A and B). Next, we converted these fibroblast lines into iPSCs using Sendai virus to overexpress the Yamanaka factors (Klf4, Oct3/4, Sox2, and c-Myc) [42]. After generating several iPSC clones from each fibroblast line, we chose one clone per patient with appropriate morphology and karyotype for downstream use and analysis (Supplementary Material, Fig. S1C). We first confirmed the expression of pluripotency markers, and their increased expression compared to the parental fibroblasts (Fig. 1B and Supplementary Material, Fig. S1D). Concurrently, we also cultured a panel of iPSC lines from unaffected individuals, three of which were previously published [43, 44], and the fourth was generated in this study. These unaffected iPSC lines are derived from healthy males of approximately similar ages as the MDS individuals at the time of tissue collection (Supplementary Material, Fig. S1A). This cohort of unaffected lines serves as the control group, as we cannot use genome editing technology to generate isogenic control cell lines per individual as could be done for disorders caused by single nucleotide variants [45]. We found that iPSCs generated from MDS probands express pluripotency markers, and we detected no RNA expression differences in *SOX2* and *NANOG* in MDS iPSCs compared to unaffected control iPSCs (Fig. 1C). Furthermore, we found that the MDS iPSCs express ~2-fold of *MECP2* RNA compared to unaffected controls (Fig. 1D). These data confirm, along with previous work [39], that *MECP2* dosage does not prevent iPSC reprogramming and that increased *MECP2* expression in MDS-derived iPSC lines is preserved.

### Characterization of *MECP2* RNA and protein expression in iPSC-derived neurons during NGN2-driven differentiation

**Figure 2:**
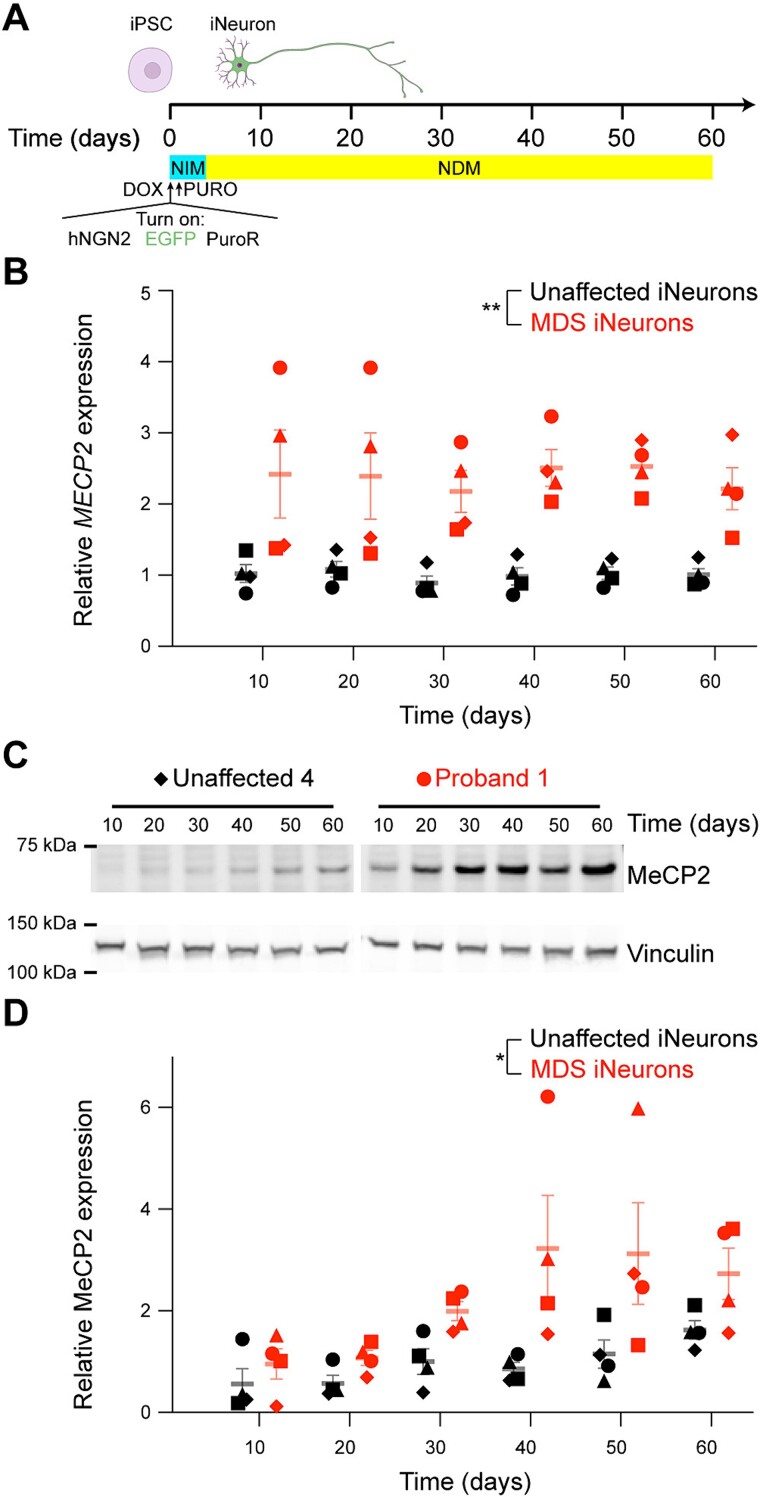
*MECP2* RNA and protein expressions increase during NGN2-directed differentiation. (A) Schematic of differentiation protocol used in this study. iPSCs are plated in neural induction media (NIM) with doxycycline to induce NGN2 expression. Non-transduced cells are selected against with puromycin treatment. After 4 days, the immature neurons are then cultured in neural differentiation medium (NDM) with doxycycline and puromycin. (B) *MECP2* RNA expression over 60 days of NGN2-directed differentiation in both unaffected or MDS neurons as measured by qPCR. (C and D) MeCP2 protein expression over 60 days of NGN2-directed differentiation as measured by western blot. (C) Representative MeCP2 protein expression over 60 days of culture for one unaffected and MDS cell line as measured by western blot. Vinculin was used as a loading control. The gap separates non-contiguous samples on the membrane. (D) Quantification of MeCP2 signal intensity normalized to Vinculin signal intensity per genotype per individual. Graphs display mean ± sem of *n* = 4 biological replicates per genotype, individuals are depicted with a separate shape. RNA and protein quantifications were normalized to the control group at day 30 in culture. Differences in gene or protein expression were assessed by two-way ANOVA (^*^P_genotype_ < 0.05, ^*^^*^P_genotype_ < 0.01).

*MECP2* RNA and protein levels are developmentally regulated and continue to postnatally increase into adulthood in both mice and humans [46, 47]. In the mouse brain, *Mecp2* RNA exhibits a mild up-regulation (< 2-fold), while MeCP2 protein exhibits a marked increase (> 5-fold) over time in the brain [47, 48]. A similar pattern was observed for *MECP2* expression in neurons derived from iPSCs using growth factor mediated differentiation [39]. Given that MeCP2 is predominantly expressed in neurons [48] and to mitigate cell type heterogeneity during differentiation [49], we engineered our iPSC lines to express doxycycline-inducible human neurogenin 2 (NGN2) (Fig. 2A). Overexpression of NGN2 pushes iPSCs towards a glutamatergic excitatory cortical neuron fate, which we will refer to as an iNeuron [50]. We confirmed that NGN2 overexpression results in the generation of Vglut1^+^ cells that are positive for the neuronal cytoskeletal proteins MAP2 and beta III tubulin in MDS patient lines (Supplementary Material, Fig. S2A) [47]. To assess the trajectory of *MECP2* expression in iNeurons, we cultured neurons for 60 days and sampled both RNA and protein expression at 10-day intervals. We first found that *MECP2* expression was increased nearly 2-fold compared to iPSCs once differentiation was started (Supplementary Material, Fig. S2B). We also found that *MECP2* RNA expression level is generally stable over 60 days in culture, with most lines exhibiting either no change or a slight increase of *MECP2* RNA during that time frame (Fig. 2B). In contrast, MeCP2 protein increases by approximately 2-fold in both genotypes, plateauing after 30–40 days in culture (Fig. 2C and D and Supplementary Material, Fig. S2C). We further found that MDS iNeurons express nearly 2-fold *MECP2* RNA compared to unaffected individuals at each time point, while MeCP2 protein differences were more variable and tended to be at least 2-fold. The difference in MeCP2 protein levels between genotypes exacerbates over time in culture (Fig. 2C and D). These data demonstrate that increased MeCP2 dosage does not prevent the differentiation of iPSCs into excitatory neurons using NGN2 overexpression and that MeCP2 protein follows a qualitatively similar pattern of up-regulation during differentiation and maturation of iNeurons as described in mouse and human brain development.

### Analysis of global gene dysregulation in MDS iNeurons

**Figure 3:**
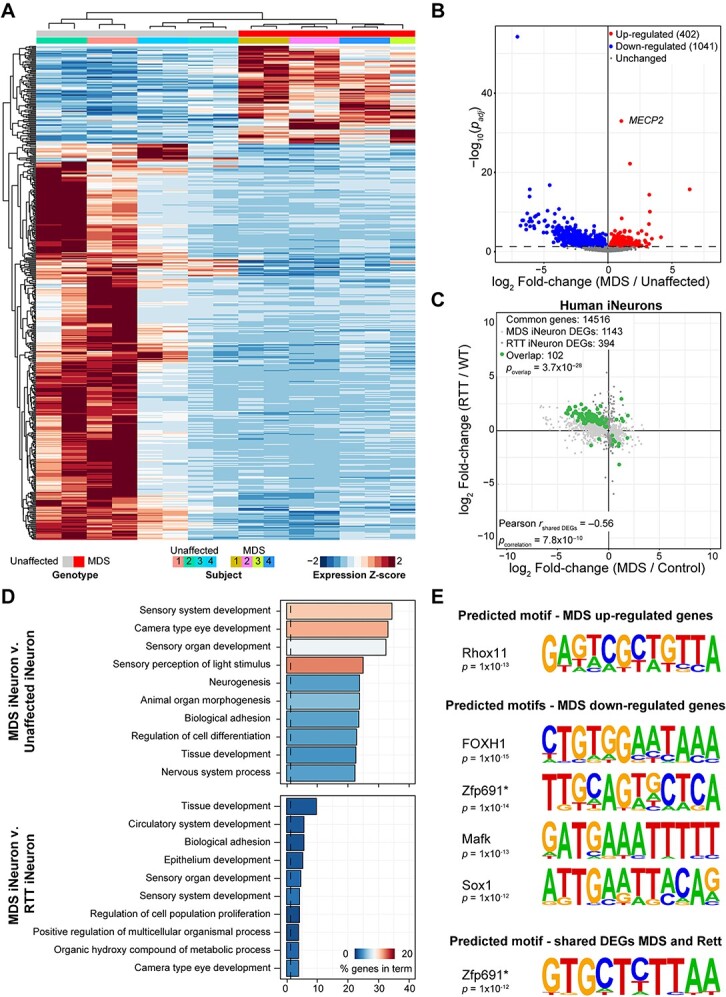
Global gene expression is altered in *MECP2* duplication syndrome iNeurons. (A) Heatmap of the Z-scored expression of the top-most significantly (*p*_adj_ < 0.005) altered transcripts. Profiles are clustered by row and column using Euclidean distance. Sample genotype and individual is denoted by the color bar above the heatmap per the key below. (B) Volcano plot denoting significant (*p*_adj_ < 0.05) gene expression changes between MDS iNeurons (*n* = 7) and unaffected iNeurons (*n* = 8). *MECP2* expression is highlighted by text. (C) Comparison of gene expression changes between MDS iNeurons and human iPSC-derived Rett syndrome (RTT) neurons using RNA-seq. Graph plotting the log_2_ fold-change between MDS iNeurons on the x-axis versus the respective log_2_ fold-change of the RTT neurons on the y-axis of the significantly (*p_adj_* < 0.05) altered genes in either genotype or shared between genotypes. The significance of overlap is shown in the upper-left corner as assessed with a Fisher’s exact test. The Pearson correlation coefficient and significance is shown in the bottom left corner. (D) Gene ontology analysis of transcriptional programs dysregulated in MDS (top panel) or shared between MDS and RTT iNeurons RNA-seq (bottom panel). Vertical line denotes *p*_adj_ = 0.05, and the color of the bar represents the percentage of genes dysregulated per total genes in each GO term. (E) Predicted transcription factor motifs of MDS disease signature using HOMER. The HOMER tool was used to search for transcription factor motifs in gene sets up-regulated in MDS iNeurons (top), down-regulated in MDS iNeurons (middle), or inversely regulated in MDS and RTT iNeurons (bottom). The best match DNA-binding protein for the identified motif is shown for those that passed false discovery (see Supplementary Material, Table S5). Asterisk denotes a best match transcription factor, Zfp691, that was also identified amongst dysregulated genes that were oppositely regulated in RTT iNeurons in panel (C). Motifs that had a *P*-value less than 1 × 10^−11^ were considered significantly enriched above background per the false positive flag from HOMER (see Methods).

Increased *MECP2* dosage has been shown to cause the dysregulation of hundreds to thousands of genes in mice [13, 14, 20, 29, 31, 51]. We sought to use our MDS iNeuron model to test if increased *MECP2* dosage similarly caused widespread gene dysregulation in human neurons and to test if this disease signature could be rescued upon normalization of MeCP2 levels using an ASO. To address these questions, we first needed to understand if ASO treatment itself caused any transcriptional changes. We cultured MDS and unaffected control iNeurons for thirty days and then continually treated cells with either a non-targeting scramble control ASO or vehicle for two weeks and performed RNA sequencing (Supplementary Material, Table S1). Encouragingly, we found scramble ASO treatment did not induce any significant gene expression changes in either genotype (Supplementary Material, Fig. S3A and B, Supplementary Material, Table S2, and Supplementary Material, Table S3). Given these results, we then pooled the vehicle-treated and scramble control ASO-treated samples to increase our power in determining the baseline gene expression signature caused by *MECP2* duplication using the DESeq2 analysis pipeline, as MeCP2-dependent gene expression changes tend to be small in magnitude [20, 52, 53]. Using the LimmaVoom pipeline to analyze the data yielded similar effect sizes between genotypes, but the variation amongst cell lines muted the *P*-values determined when compared to DESeq2. Thus, we moved forward in our studies using the results tabulated by DESeq2 (Supplementary Material, Tables S1–S3) [53–55]. Global analysis of gene expression changes identified that the samples were separated by genotype, and that replicate transcriptomes from individuals clustered together (Fig. 3A). The variability between cell lines was captured by principal component analysis, with each line occupying separate areas in space (Supplementary Material, Fig. S3C). Next, from the global transcriptional dysregulation, we identified approximately 1500 dysregulated genes between MDS and unaffected control neurons (*p*_*adj* <_ 0.05), with nearly 66% of these genes down-regulated (Fig. 3B). Previous work had implicated loss of MeCP2 in regulating overall transcription and translation in RTT neurons as contributing to gene downregulation [56]. However, we did not observe any significant differences in the levels of key components of the AKT/mTOR pathway, nor did we detect an enrichment in the dysregulation of polymerase or ribosomal protein genes (Supplementary Material, Fig. S4 and Supplementary Material, Table S1), suggesting other mechanisms may be driving gene dysregulation in MDS iNeurons. Nonetheless, taken together, these data demonstrate that there are transcriptional differences between MDS and unaffected control iNeurons.

The loss of MeCP2, like the increase of *MECP2* dosage, causes the dysregulation of hundreds to thousands of genes [19]. Additionally, multiple labs have shown that several transcripts are regulated in opposing directions by either the loss or gain of MeCP2 in mice [20, 29–31, 51]. To capitalize on this phenomenon to identify a list of MeCP2-sensitive genes, we compared the genes dysregulated in MDS iNeurons in our study with genes dysregulated in a previously published RNA-sequencing dataset generated in RTT iNeurons [37]. Interestingly, MDS iNeurons shared a significant overlap of 102 dysregulated genes with RTT iNeurons (Supplementary Material, Table S4). Furthermore, the directionality of dysregulation was significantly anticorrelated (*r* = −0.57, *P* < 0.05) (Fig. 3C). To characterize programs of gene expression regulated by MeCP2 in human neurons, we performed gene ontology analysis of the differentially expressed genes between 1) MDS and unaffected lines or 2) shared between MDS and RTT iNeurons. In both comparisons, the top terms were related to tissue patterning and development (Fig. 3D). We then further investigated if these programs of genes regulated by MeCP2 shared any potential candidate transcriptional regulators using the HOMER transcription factor motif enrichment tool (see Methods) [57]. For genes up regulated in MDS lines, we detected just one motif that was significant above false discovery for mouse Rhox11 (human protein ortholog is RHOXF1). For genes downregulated in MDS lines, we identified four motifs that were significantly enriched above false discovery for FOXH1, Zfp691 (human protein is ZNF691), Mafk (human protein is MAFK), and Sox1 (human protein is SOX1) (Fig. 3E and Supplementary Material, Table S5). Intriguingly, a separate Zfp691/ZNF691 motif was also enriched when considering the genes jointly dysregulated amongst the RTT and MDS lines with motifs identified in ~10% of the input genes, pointing to a potential candidate downstream of MeCP2 that may regulate the disease signature. Taken together, these data suggest that the transcriptome of human iPSC-derived neurons is reciprocally sensitive to MeCP2 protein levels, particularly in a subset of genes related to development, akin to what has been observed in mouse models of MeCP2-related disorders.

### Pharmacodynamics of antisense oligonucleotide treatment *in vitro*

**Figure 4:**
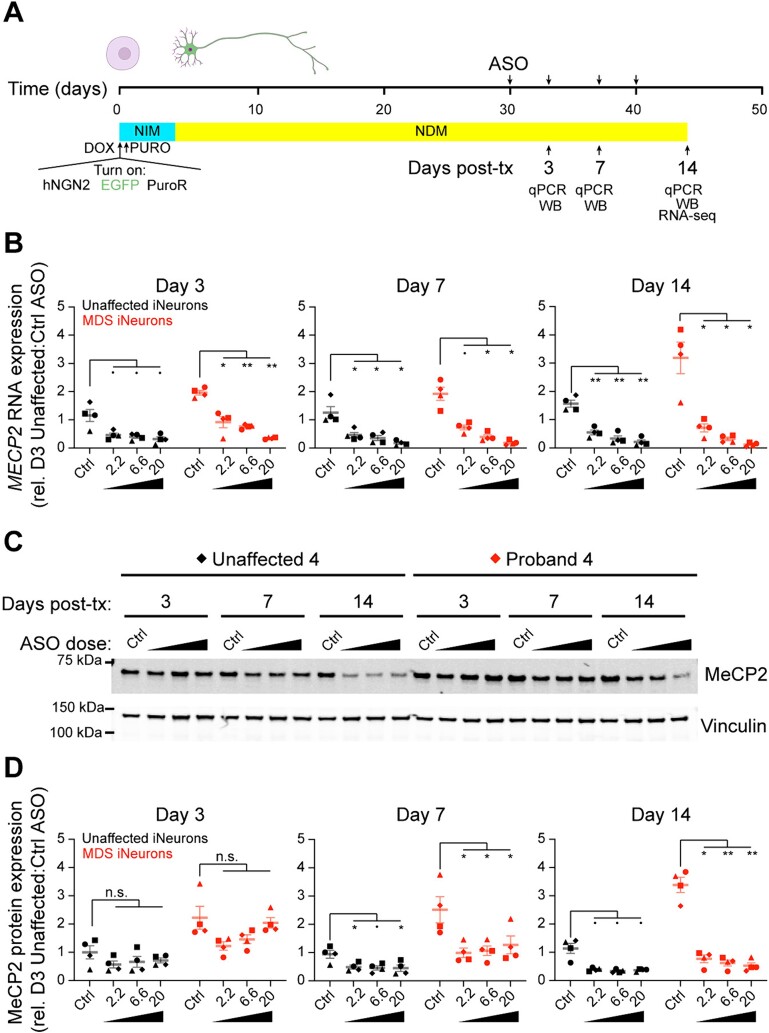
Antisense oligonucleotide treatment acutely reduces both *MECP2* RNA expression and MeCP2 protein levels in culture. (A) Schematic of differentiation and ASO treatment protocol. ASOs in three doses (in μM) were introduced into the media starting 30 days after culture and supplemented with fresh ASO at 3, 7, and 10 days after the first dose. Control scramble ASO was used at the maximum dose. RNA and protein lysates were collected 3, 7, and 14 days after first dose of ASO. (B) *MECP2* RNA expression after ASO treatment as measured by qPCR. (C and D) MeCP2 protein expression after ASO treatment as measured by western blot. (C) Representative MeCP2 protein expression during ASO treatment for one unaffected and MDS cell line as measured by western blot. Vinculin was used as a loading control. (D) Quantification of MeCP2 signal intensity normalized to Vinculin signal intensity per genotype per individual. Graphs display mean ± sem of *n* = 4 biological replicates per genotype, individuals are depicted with a separate shape. Data are normalized to RNA or protein quantification of the unaffected neurons treated with scramble control ASO at day three of treatment. Data are displayed for one *MECP2* ASO sequence (see Supplementary Material, Fig. S5 for data using a second *MECP2* ASO sequence). Data were analyzed by two-way ANOVA and Sidak’s multiple comparisons test to scramble controls (⋅ *P* < 0.1, ^*^P < 0.05, ^*^^*^P < 0.01).

We next tested if treatment with an ASO that targets *MECP2* for RNase H1-mediated degradation (*MECP2* ASO) in human neurons can normalize MeCP2-induced gene expression similar to what occurs in MDS mice treated with *MECP2* ASO [13, 14]. We have extensively described the pharmacodynamics of *MECP2* ASO treatment in mice [14], but the dynamic changes in MeCP2 protein levels after ASO treatment have not been measured in human neurons. We differentiated neurons for thirty days and then treated these neurons with either a scramble ASO or two independent *MECP2* ASOs at three different doses (2.2, 6.6, and 20 μM). Fresh ASO was supplemented at each half-media change (three, seven, and ten days after first treatment). We then collected samples at three, seven, and fourteen days after the first ASO treatment and measured *MECP2* RNA and protein (Fig. 4A). Here, we found that *MECP2* RNA was acutely and significantly (*P* < 0.05) reduced to below 50% within three days of treatment in unaffected control neurons. In MDS neurons, we observed a dose-dependent response, where the maximum 20 μM dosage of ASO reduced *MECP2* RNA to 35% of scramble ASO treated MDS neuron expression. The knockdown of *MECP2* remained at a similar level at day seven and fourteen (Fig. 4B). The reduction of MeCP2 protein lagged compared to the knockdown of *MECP2* RNA, as we have observed in mice [14]. We observed a trend towards a reliable decrease of protein three days after ASO treatment, and this decrease was significant (*P* < 0.05) by seven days after ASO treatment in both genotypes. At this time point, the MeCP2 protein levels in MDS neurons treated with *MECP2* ASO were similar to MeCP2 protein levels in unaffected control neurons. By day fourteen, we observed a robust decrease in MeCP2 protein levels in both genotypes. At the highest dosage, MeCP2 levels were reduced to below 50% of unaffected control neurons treated with scramble ASO in both genotypes (Fig. 4C and D). The reduction, kinetics, and dose–response of *MECP2* RNA and protein results were qualitatively repeated with a second *MECP2* targeting ASO (Supplementary Material, Fig. S5A–C). These data demonstrate that ASO treatment is effective in knocking down *MECP2* expression in human neurons and that reduction in MeCP2 protein lags behind *MECP2* RNA expression. Thus, we can model ASO treatment in MDS iNeurons to further assess if ASO treatment restores normal gene expression.

### Qualitative rescue of gene expression in MDS iNeurons after ASO treatment

Abnormal global gene expression downstream of increased *MECP2* dosage is dynamically rescued upon normalization of MeCP2 protein in mice by ASO treatment, with peak molecular rescue occurring at fourteen days after ASO treatment [14]. These gene expression changes preceded behavioral and phenotypic rescue, suggesting normalizing gene expression is a key read out of ASO treatment efficacy. To test if rescue of gene expression after ASO treatment occurs in human MDS iNeurons, we performed bulk RNA-sequencing of both unaffected control and MDS iNeurons after fourteen days of ASO treatment at the two highest doses of both *MECP2* ASOs (Fig. 4A). We found that *MECP2* ASOs had a subtle global effect on gene expression in both genotypes with few genes being significantly altered (*p_adj_* < 0.05) compared to scramble ASO, which can be at least partially attributed to the variation amongst the cell lines, the low magnitudes of gene expression changes, and multiple comparison correction (Supplementary Material, Tables S2 and S3). Thus, we focused on the genes robustly dysregulated in MDS iNeurons at baseline (Fig. 3A). We qualitatively assessed the directionality of gene expression using two methods: 1) by using principal component analysis and 2) by ranking the relative expression in unaffected control cell lines, *MECP2* ASO-treated unaffected control cell lines, MDS cell lines, and *MECP2* ASO-treated MDS cell lines per gene. In principal component space, we observed that samples clustered most similarly within individual in both genotypes. In unaffected control samples, we first noticed a significant (*P* < 0.05) shift of ASO-treated samples down principal component 2. This shift was generally preserved, though not statistically significant, in MDS ASO-treated samples as well, bringing the MDS ASO-treated samples towards the gene expression signature of the unaffected controls, suggesting at least some genes may be partially rescued in the MDS-disease signature upon ASO treatment (Supplementary Material, Fig. S6A). Next, we examined the rank order of genes within the disease signature amongst experimental groups and clustered this ranked dataset and found that the unaffected control lines clustered separately from the MDS cell lines. Additionally, the *MECP2* ASO-treated MDS lines clustered in between the unaffected and MDS lines in both *MECP2* ASO sequences (Supplementary Material, Fig. S6B and C), matching our expectation of global gene expression signatures based on *MECP2* dosage. These data suggest that at least a subset of genes was qualitatively and partially rescued in expression in the MDS neurons upon *MECP2* ASO treatment.

**Figure 5:**
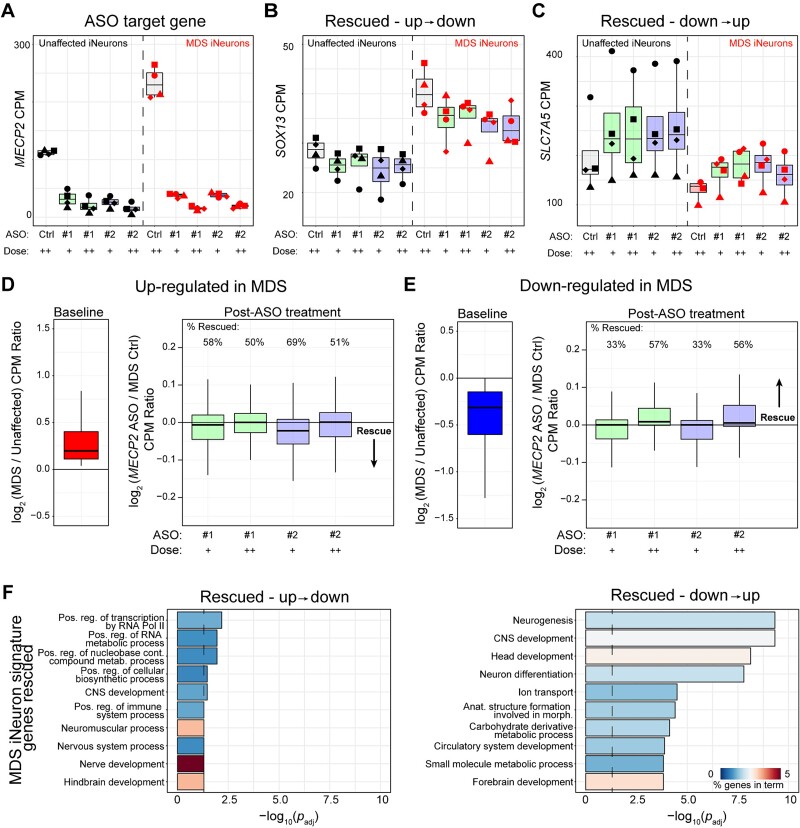
Qualitative rescue of gene expression after ASO treatment in *MECP2* duplication syndrome iNeurons. (A—C) Counts per million for the ASO-target gene *MECP2* (A), *SOX13* (B), and *SLC7A5* (C) in unaffected and MDS lines for control scramble ASO (ctrl) or each anti-*MECP2* ASO sequence across two doses (+ is 6.6 μM dose, ++ is 20 μM dose, control scramble ASO used at 20 μM dose). *SOX13* and *SLC7A5* are example genes that are rescued in ASO-treated MDS iNeurons and also aberrantly dysregulated in ASO-treated unaffected control neurons. Graphs display boxplots of *n* = 4 biological replicates per condition, individuals are depicted with a separate shape. (D and E) Rescue of genes that are up-regulated (D) or down-regulated (E) at baseline in MDS iNeurons compared to unaffected iNeurons. The boxplots depict the distribution of the log_2_ ratio of the average counts per million between either MDS and unaffected neurons (left inset panels) or anti-*MECP2* ASO treated and MDS control (scramble ASO and naïve) neurons for a single gene. Genes with a ratio above 0 are up-regulated in the numerator, while below 0 are down-regulated in the numerator (right inset panels). Genes that were up-regulated in MDS lines at baseline and that were qualitatively reduced towards unaffected control levels after ASO treatment fall below 0 on the right inset panel in (D). Genes that were down-regulated in MDS lines at baseline and that were qualitatively increased towards unaffected control levels after ASO treatment fall above 0 on the right inset panel in (E). The percent of genes trending towards rescue, as depicted by the direction of the arrow, for either up- or down-regulation are shown above each ASO treatment condition. (F) Gene ontology analysis of transcriptional programs rescued in MDS lines. Programs are split by up- and down-regulated genes when identifying ontologies. Vertical line denotes *p*_adj_ = 0.05, and the color of the bar represents the percentage of genes dysregulated per total genes in each GO term in both graphs.

A closer examination of the genes dysregulated at baseline between MDS and control lines identified genes that were modulated by ASO treatment (Fig. 5A–C). *MECP2* is robustly down-regulated in both unaffected control and MDS lines treated with *MECP2* ASO, confirming our qPCR results (Fig. 4B, Fig. 5A, and Supplementary Material, Fig. S5A). We also observed genes that were consistently down-regulated upon ASO treatment in both genotypes. For example, *SOX13* is normally up-regulated in MDS lines, and *MECP2* ASO treatment restores this gene back to unaffected control levels and causes a down-regulation of *SOX13* in *MECP2* ASO-treated unaffected control lines (Fig. 5B). We also observed reciprocal cases; for example, *SLC7A5* is normally down-regulated in MDS lines, and *MECP2* ASO treatment restores this gene back to unaffected control levels and causes an up-regulation of *SLC7A5* in *MECP2* ASO-treated unaffected control lines (Fig. 5C). We also observed genes that were dysregulated in MDS lines compared to unaffected control but were not modulated by ASO treatment in either genotype. Some examples include genes implicated to be regulated by *MECP2*, like *BDNF, SSTR1*, and *BMPER* (Supplementary Material, Fig. S7A and B) [58]. Given these examples of varying effects of ASO treatment on individual genes, we examined the global extent of rescue of the genes dysregulated in MDS iNeurons and discovered that ~55% of the up- and ~45% down-regulated genes were qualitatively and partially rescued towards unaffected control expression after ASO treatment (Fig. 5D and E). We then examined the genes jointly dysregulated between MDS and RTT iNeurons and identified that a similar percentage of this subset of genes was qualitatively rescued by *MECP2* ASO treatment in the MDS neurons (Supplementary Material, Fig. S6D). Lastly, we examined genes that were qualitatively aberrantly altered in ASO-treated unaffected control lines in an opposite direction of the MDS disease signature (Supplementary Material, Fig. S6E). This subset of genes represents those that are responsive to acute decreases in MeCP2 levels. Of this subset, nearly ~70% of the up- and ~65% of the downregulated genes were qualitatively rescued. Overall, we identified approximately 300 genes that were altered in either RTT iNeurons or ASO-treated unaffected iNeurons that were qualitatively rescued in *MECP2* ASO-treated MDS iNeurons, comprising nearly ~30% of the overall disease signature modulated.

To understand the gene programs that are sensitive to MeCP2, we performed gene ontology analysis of the genes that were qualitatively rescued. We found that gene programs involved in neurogenesis and central nervous system development were significantly enriched in both up- and down-regulated genes rescued in MDS lines (Fig. 5F). Taken together, these data demonstrate that gene expression is modulated in human neurons upon an acute reduction in *MECP2* dosage independent of genotype, that a subset of dysregulated genes are partially and qualitatively rescued with ASO treatment in human MDS neurons, and that these genes are involved in neural differentiation and development.

### ASO treatment partially rescues aberrant neuronal morphology in MDS iNeurons

**Figure 6:**
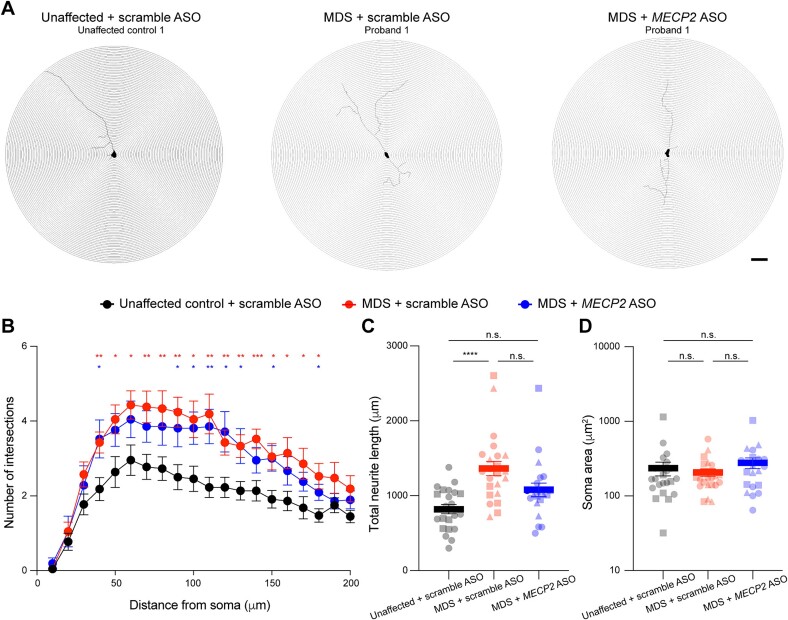
Two weeks of *MECP2* ASO treatment leads to mild improvements in neuronal morphological deficits in *MECP2* duplication syndrome iNeurons. (A) Representative neuronal traces from individual, fluorescently tagged iNeurons. The skeletonized image was generated in Neurolucida and used for Sholl analysis. Scale bar is 100 μm. (B) Quantification of neurite intersections at concentric lengths away from the soma size. (C) Quantification of total neurite length per analyzed neuron. (D) Quantification of soma area per analyzed neuron. In total 23 neurons from unaffected control lines treated with 20 μM scramble ASO (15 from unaffected control 1, 8 from unaffected control 2), 22–23 neurons from MDS lines treated with 20 μM scramble ASO (8 from Proband 1, 7–8 from Proband 2, and 7 from Proband 3), and 21 neurons MDS lines treated with 20 μM *MECP2* ASO#1 (9 from Proband 1, 5 from Proband 2, and 7 from Proband 3) were analyzed using the Neurolucida software (see Methods). Data were analyzed by mixed effects two-way ANOVA with post-hoc multiple comparisons test (B) or one-way Brown-Forsythe and Welch ANOVA with post-hoc multiple comparisons test (C and D) with ^*^P < 0.05, ^*^^*^P < 0.01, ^*^^*^^*^P < 0.001, and ^*^^*^^*^^*^P < 0.0001.

In addition to transcriptional dysregulation, increased *MECP2* dosage has been shown to cause abnormal neuronal morphology, notably causing increased dendritic arborization [39, 59]. To test if ASO treatment normalized neuronal morphology, we repeated our differentiation and ASO treatment paradigm (Fig. 4A) in chamber slides (*n* = 2 unaffected control lines and *n* = 3 MDS lines). We further sparsely labeled single neurons within each culture using an adeno-associated virus (AAV) driving expression of the fluorescent protein tdTomato (Fig. 6A) (see Methods). We imaged multiple fixed neurons per condition and measured dendritic complexity using Sholl analysis, which quantifies the number of neurite intersections at distance intervals from the soma. We observed that scramble ASO-treated MDS iNeurons exhibited significantly (*P* < 0.05) increased dendritic complexity compared to scramble ASO-treated unaffected neurons, reproducing the previously reported phenotype (Fig. 6B) [39, 59]. Next, we assessed morphology in MDS iNeurons treated with the highest dose of *MECP2* ASO (20 μM) and found they displayed qualitatively reduced dendritic complexity than the scramble ASO-treated MDS iNeurons. Interestingly, while scramble-treated MDS iNeurons displayed a significant (*P* < 0.05) increase in process intersections at 75% of the distances away from the soma, ASO-treated MDS iNeurons displayed a significant (*P* < 0.05) increase in only 40% of the distances, being indistinguishable from scramble treated unaffected neurons at 60% of the assessment points (Fig. 6B). This subtle rescue is also reflected when considering the total neurite length as a feature of neuronal complexity. Total neurite length was significantly increased (*P* < 0.0001) in scramble ASO-treated MDS iNeurons compared to scramble ASO-treated unaffected control iNeurons, and *MECP2* ASO treatment led to a reduction of total neurite length in MDS neurons towards the total neurite length in scramble ASO-treated unaffected control iNeurons (Fig. 6C). We detected no baseline difference, nor change upon ASO treatment, in soma size between unaffected control and MDS iNeurons (Fig. 6D). These data indicate that a two-week treatment of *MECP2* ASO is sufficient to begin to drive improvements, but not completely rescue neuronal morphology in MDS iNeurons.

## Discussion

The use of patient iPSC-derived neurons has enabled us to model and examine neurodevelopmental diseases in a human neuronal context. In this study, we generated excitatory cortical neurons from four MDS probands and used these cellular avatars to test the effects of using *MECP2* ASOs on gene expression in human neurons. We found that *MECP2* ASOs effectively and rapidly reduce *MECP2* RNA and MeCP2 protein, leading to a downstream rescue of global gene expression. These experiments revealed key gene expression programs acutely and sensitively regulated by MeCP2 that were not previously described in studies using iPSC-derived neurons modeling MeCP2-related disorders [37, 39, 40, 60].

MeCP2 protein levels increase during development in mice and humans and this upregulation coincides with the maturation of the brain [30]. We observed a similar trajectory in NGN2-differentiated iPSC-derived neurons from both control and MDS genotypes (Fig. 2D). These results have two important implications. First, MeCP2 protein levels track with the increase in neuronal maturity in culture. This trajectory of MeCP2 protein levels is supported by our discovery that MeCP2 sensitively and reciprocally regulates genes involved in neurogenesis and neural development in human neurons (Fig. 3D). Second, understanding the timeline of endogenous upregulation of MeCP2 protein expression in culture was critical in designing our ASO treatment experiments. For instance, if we had performed ASO treatment at day ten in culture, when MeCP2 protein is lowly expressed (Fig. 2D), we may have had difficulties in assessing ASO efficacy or in observing gene expression alterations from *MECP2* ASO treatment. Thus, characterizing the regulatory trajectory of protein expression for a gene of interest is critical when modeling neurological diseases in iPSC-derived neurons.

Much of our understanding of connecting *MECP2* dosage and gene expression comes from transcriptional profiling of mouse models of RTT and MDS. These studies have importantly revealed that a subset of genes is oppositely regulated in mouse models of RTT and MDS [20, 29, 31, 51]. In this study, we uncovered a signature of genes that is inversely dysregulated between RTT and MDS iNeurons, showing that this principle of reciprocal gene regulation is also observed in a human neuronal context. Moreover, many of these genes were acutely and aberrantly altered in unaffected control iNeurons treated with *MECP2* ASOs, further demonstrating that gene expression is responsive to MeCP2 protein levels in human neurons. Importantly, these two independent contexts of MeCP2 loss-of-function identified a signature of genes that is responsive to an acute or constitutive downregulation of MeCP2 levels or function. As we develop newer therapeutics that modulate MeCP2 stability or levels [61], the panel of genes identified in this study can be now added to the repertoire of molecular and physiologic readouts to assess treatment efficacy *in vitro*. Additionally, the genes responsive to MeCP2 dosage can also reveal new insights into disease pathogenesis [62].

Here, we showed that gene expression programs revolving around neurogenesis are dysregulated in MDS iNeurons and are potentiated by ASO treatment. For example, *SOX13* has been implicated in the maturation and specification of neurons in the developing mouse [63]. Further investigation will be required to understand how the baseline increase in *SOX13* expression in MDS iNeurons affects neuronal maturation *in vitro.* Additionally, we observed that *SLC7A5* is down-regulated in MDS iNeurons and increased after ASO treatment in both MDS and unaffected control iNeurons. *SLC7A5* is an important amino acid transporter that is expressed during neural development whose loss has been associated with neurodevelopmental phenotypes [64, 65]. The importance of *SLC7A5* expression and function has been primarily established for brain endothelial cells [65], and our data suggest that *SLC7A5* expression is regulated by MeCP2 in human neurons and warrants further investigation. Our analyses also suggest that ZNF691 may be a transcriptional modulator downstream of MeCP2 dosage in cultured human neurons. The motif for ZNF691 was enriched in the gene set down-regulated in MDS and the gene set shared and inversely regulated in RTT and MDS iNeurons. Very little is known about the transcriptional regulators downstream of MeCP2 that lead to specific gene expression changes. Further work will be needed to assess if ZNF691 could be one of those factors that functionally explains at least a subset of gene expression changes downstream of MeCP2.

Two weeks of *MECP2* ASO treatment qualitatively modulated a subset of the genes dysregulated in MDS neurons towards the levels in unaffected controls. Furthermore, we observed only a partial and subtle rescue in the MDS lines treated with *MECP2* ASO. This may be due to the lag in time between MeCP2 protein levels and modulation of MeCP2-dependent gene expression, which we have previously described using mouse models [13, 14]. While two weeks of *MECP2* ASO treatment was sufficient to strongly rescue gene expression in MDS mice, the time lag may be different in human neurons *in vitro* or humans *in vivo*. It is possible that longer treatment with ASO could yield a greater and quantitative extent of gene expression rescue. Gene expression rescue precedes phenotypic rescue in mice by nearly a month, but whether the timing of phenotypic rescue is similar in human neurons remains unknown. Here, we observed a mild improvement in neuronal morphology after two-weeks of ASO treatment. This was a surprise because the gene expression rescue was only partial at this time point. Like with gene expression improvements, a longer treatment with ASO could yield a greater extent of functional and morphological rescue *in vitro*. An understanding of the specific timing and sequence of molecular and phenotypic rescue downstream of restored MeCP2 protein levels, particularly considering the timing as we understand it in mouse models, will be beneficial to benchmark realistic evaluations of treatment efficacy in *MECP2* ASO clinical trials.

A limitation of this study is in the use of two-dimensional neuronal cultures generated through NGN2-directed differentiation. Although NGN2-directed differentiation drives iPSCs towards a cortical, excitatory fate [50], a recent single-cell study revealed heterogeneity of the specific neuronal and maturity markers of neurons within a given NGN2-driven culture [66]. Thus, the effects of genetic background and/or ASO treatment could be affecting an individual type of neuron more than others, and this may be contributing to the cell line transcriptomic heterogeneity and overall subtle and qualitative extent of gene expression changes in response to ASO treatment. Driving increased neuronal maturity using three-dimensional organoid cultures could potentially mitigate these caveats and enhance the effects of ASO treatment on gene expression [40, 67]. Future work using single-cell transcriptomics, in conjunction with three-dimensional organoid cultures, will help to determine the full and quantitative extent of the benefits of ASO therapy in heterogeneous MDS human neurons.

While mouse models have been critical in establishing the proof-of-principle for ASO therapy in MDS, these studies have the caveat that each mouse used was genetically identical harboring one additional copy of human *MECP2*; however, MDS CNVs encompass many genes. In this study, we treated patient iPSC-derived neurons with ASOs to perturb only *MECP2* expression in the context of CNVs spanning multiple genes, which revealed that rescuing MeCP2 levels alone leads to a partial and qualitative rescue of gene expression. As the minimal genomic region of overlap in MDS contains *MECP2* and *IRAK1*, future studies perturbing *IRAK1* individually will determine if the remaining molecular signature is regulated by *IRAK1* or is secondary to the CNV. Our study establishes a paradigm of using acute perturbations to gene dosage to determine the contribution of individual genes to molecular phenotype in CNV disorders where the minimal region of overlap involves multiple genes, like 16p11.2 duplication/deletion, Williams, and DiGeorge syndromes.

## Materials and methods

### Fibroblast culture

Four male individuals diagnosed with *MECP2* duplication syndrome consented to give a skin biopsy for the purposes of generating induced pluripotent stem cell lines after obtaining written, informed consent as approved by the Institutional Review Board at Baylor College of Medicine. One unaffected male individual also consented for a skin biopsy and iPSC cell generation. The age at time of skin biopsy and gender of these individuals is described in Supplementary Material, Fig. S1A. Skin biopsies were processed into fibroblast cell line culture by the IDDRC Tissue Culture Facility Core at Baylor College of Medicine. Briefly, skin biopsies were rinsed in DBPS and placed at the bottom of a 6 cm tissue culture dish. Biopsies were minced between two sterile scalpel blades. Alpha-MEM +10% fetal bovine serum (FBS) + 1× Penicillin–Streptomycin was placed on top, and minced material was cultured at 37°C in 5% CO_2_ and 5% O_2_. Cultures were monitored for fibroblast cell outgrowth. When fibroblast cells sufficiently expanded, the culture was trypsinized and expanded in T25 and T75 flasks until ready for further processing.

### Copy number variation analysis by array comparative genome hybridization (aCGH)

The genomic characterization and CNV breakpoint junction mapping of probands 1,2, and 4 were performed in a previous study [41]. aCGH analysis for proband 3 was performed in this study using DNA extracted from fibroblasts using the Gentra Puregene kit (Qiagen #158043), and ethanol precipitated prior to sample preparation. We evaluated copy-number changes in chromosome X using a custom 4 × 180 K tiling-path oligonucleotide microarray spanning the entirety of chromosomes X and Y, including the *MECP2* locus (Agilent Technologies microarray AMADID #086099). Array design information is described in Grochowski *et al*. [68]. Array was run according to the manufacturer’s protocol (Agilent Oligonucleotide Array-Based CGH for Genomic DNA Analysis, version 7.3, Agilent Technologies) with modifications as previously described [69]. Coordinates for each CNV observed along those chromosomes were annotated. The genomic contexts where breakpoint junctions occur were investigated using UCSC Genome Browser GRCh37/hg19 Assembly (http://genome.ucsc.edu) [70] for information about the presence of repeats, low-copy repeats, and pseudogenes.

### Generation, quality control, and culture of induced pluripotent cells (iPSCs)

Skin fibroblasts collected as described above were reprogrammed into iPSCs by the Baylor College of Medicine Human Stem Cell Core (HSCC) using the Cyto-Tune iPS 2.0 Sendai Reprogramming Kit (ThermoFisher #A16517). Fibroblasts were infected with non-integrating Sendai viruses expressing OCT4, SOX2, KLF4 and C-MYC. At least one colony per re-programming was manually selected and expanded as a clonal iPSC population. iPSCs were cultured and expanded under feeder free conditions using hESC-qualified Matrigel (Corning #354277) and TeSR-E8 (Stemcell Technologies #05990) media or mTeSR1 media (Stemcell Technologies #85850). iPSC lines were karyotyped by the MD Anderson Cytogenetics and Cell Authentication Core. One iPSC line per individual was selected based on normal karyotype and superior iPSC colony morphology. iPSC lines were passaged using ReLeSR (Stemcell Technologies #100-0483) for at least 15 passages before preparing lines for neuronal differentiation. Cultures were regularly tested for mycoplasma contamination.

### Lentivirus production

Lentiviral particles encapsulating vectors encoding rtTA (rtTA-N144; Addgene #66810) and doxycycline-inducible hNGN2 (pLV-TetO-hNGN2-eGFP-Puro; Addgene #79823) were produced as previously described [47]. Briefly, HEK 293FT cells were transfected with the above plasmids and lentiviral packaging plasmids psPAX2 (Addgene #12260) and pMD2.G (Addgene #12259) using the jetPRIME transfection reagent (Polyplus Transfection #114-15) according to the manufacturer’s protocol. Lentiviral supernatant was collected at 24- and 48-hours post-transfection and concentrated overnight at 4°C using the Lenti-X Concentrator reagent (Takara #631231) according to the manufacturer’s protocol.

### Neuronal differentiation

iPSCs were co-infected with rtTA and hNGN2 viral particles and allowed to recover. iPSC cultures were then dissociated using Accutase (Sigma Aldrich #A6964) and counted before plating at a density of 100 000 cells per 24-well plate well. Cells were plated in neural induction media (DMEM/F12:Neurobasal (1:1) with 1× B27, 1× N2, and 2 nM Glutamax) with Y-27632 10 μM ROCK inhibitor (Selleck Chemicals #S1049) and doxycycline (1 μg/ml) for 24 h. Media was replaced with fresh neural induction media with doxycycline (1 μg/ml) and puromycin (1 μg/ml) and replaced daily with same formulation for three days. After three days, media was changed to neural differentiation media (Neurobasal with 1× B27, 2 mM Glutamax, 20 ng/ml BDNF, 10 ng/ml GDNF, 10 ng/ml NT-3, 100 μM db-cAMP, and 200 μM ascorbic acid) with doxycycline (1 μg/ml) and puromycin (1 μg/ml). The media was half-changed every 3–4 days with fresh neural differentiation media with doxycycline (1 μg/ml) and puromycin (1 μg/ml) for the duration of the experiment.

### Immunofluorescence

For all immunofluorescence experiments, cells were seeded in Ibidi 8-well chamber slides (Ibidi # 80841) coated with Matrigel as described above. iPSCs were seeded at 1000 cells per chamber, iNeurons were seeded at 5000 cells per chamber. Cells were fixed with 4% paraformaldehyde in PBS for 15 min at room temperature. iPSCs were stained for pluripotency markers using the Human Pluripotent Stem Cell 3-Color Immunohistochemistry kit (R&D Systems #SC021) according to the manufacturer’s protocol. iNeurons were washed three times in PBS, permeabilized in 0.3% Triton-X in PBS for 10 min at 4°C, washed one time in PBS, incubated in blocking buffer (PBS + 1× Roche Western [MilliporeSigma #11921673001] + 0.3% Triton-X) for 1 h at room temperature, incubated in primary antibody diluted in blocking buffer overnight at room temperature, washed three times in PBS, incubated in secondary antibody diluted in blocking buffer for 2 h at room temperature, washed three times in PBS, counterstained with DAPI (300 nM in PBS), and washed one time in PBS. Cultures remained in PBS until imaged using a Zeiss LSM 710 confocal microscope. The following primary antibodies and antibody dilutions were used to stain the iNeuron cultures: anti-Vglut1 (mouse monoclonal CL2754, MilliporeSigma #AMAB91041, RRID: AB_2665777; 1:200 dilution); anti-beta III tubulin (mouse monoclonal 2G10, Sigma #T8578, RRID: AB_1841228; 1:500 dilution); anti-MAP2 (chicken polyclonal, abcam #ab5392, RRID: AB_2138153; 1:1000 dilution). The following secondary antibodies and antibody dilutions were used to stain the iNeuron cultures: anti-mouse IgG (H + L) Highly Cross-Adsorbed AlexaFluor 555 (goat polyclonal, ThermoFisher #A-21424, RRID: AB_141780; 1:200 dilution) and anti-chicken IgY (H + L) AlexaFluor 647 (goat polyclonal, ThermoFisher #A-21449, RRID: AB_2535866; 1:200 dilution).

### Antisense oligonucleotide (ASO) treatment

Ionis Pharmaceuticals designed and synthesized the *MECP2* and scramble control ASOs as previously described [13, 14]. The ASOs consisted of 20 chemically modified nucleotides [2′-*O*-(2-methoxyethyl) (MOE) gapmer] with a central gap region of 10 deoxynucleotides that was flanked on both its 5′ and 3′ sides by five MOE-modified nucleotides. The ASO backbone included phosphodiester (PO) and phosphorothioate (PS). From 5′ to 3′ the linkages are 1-PS, 4-PO, 10-PS, 2-PO, and 2-PS. The sequence of the scramble ASO is: 5′-CCTATAGGACTATCCAGGAA-3′; *MECP2* ASO #1: 5′-TGGCACTTTCTCAGACTGTT-3′; and *MECP2* ASO #2: 5′-GTTCAATATGTCATCCGAAG-3′. ASOs were added directly to the media to achieve a final concentration as denoted in the text or figure. ASOs were added at each half-media change to achieve the same final initial concentration. Blank media with no supplements was used as a vehicle control.

### Sholl analysis of neuronal morphology

For Sholl analysis, iNeurons were seeded at 6000 cells per chamber in Ibidi 8-well chamber slides (Ibidi # 80841) coated with Matrigel and differentiated as described above. After 30 days of differentiation in culture, 20 μM of scramble control ASO or *MECP2* ASO was added to each well and refreshed with each media change to keep the concentration of ASO in the culture same. ASO treatment was continued for 2 weeks and one-week post-ASO treatment, iNeurons were infected with AAV9-TdTomato (stock concentration 1.96 × 10^8^ gc/ml) at an MOI of 10 and incubated for 3 days for sparse infection for Sholl analysis. On day 4, media was replaced with fresh NDM and fluorescence was monitored. One week after AAV9-TdTomato infection and two weeks of continuous ASO treatment, the neurons were fixed in 4% paraformaldehyde (PFA) and stained with DAPI (Invitrogen, D1306) at a final concentration of 300 nM by incubating for 5 min followed by three 10-min washes with 1× TBS. Individual neurons were imaged on Zeiss LSM 710 confocal microscope. Tracings were done on the Neurolucida software using the user-guided setting and soma area was measured on ImageJ.

### RNA extraction and qRT-PCR

RNA from cell cultures was harvested using either the Qiagen miRNeasy (Qiagen #217004) or Qiagen RNeasy (Qiagen #74104) according to the manufacturer’s protocol, including on-column DNase digestion according to the manufacturer’s protocol (Qiagen #79254). From the purified RNA, 1–3 μg total RNA was used to perform reverse transcription cDNA synthesis using the M-MLV reverse transcriptase kit with random hexamer primer (Invitrogen #2802013) according to the manufacturer’s protocol. qRT-PCR was performed using a CFX96 Real-Time System (Bio-Rad) using PowerUp SYBR Green Master Mix (ThermoFisher #A25741), 0.4 μM forward and reverse primers (Table 1), and 1:20 dilution of cDNA. The following cycling conditions were used: 95°C for 5 min, 39 cycles of 95°C for 11 s, 60°C for 45 s, plate read, a final melt of 95°C, and melt curve of 65–95°C at +0.5°C increments. The specificity of the amplification products was verified using melt-curve analysis. The Ct values were calculated with the Bio-Rad CFX Maestro Software, and relative gene expression was calculated using the DDCt method using one of the following housekeeping genes for normalization: *HPRT* (Fig. 1), *GAPDH* (Fig. 2 and Supplementary Material, Fig. S2), or *PPIA* (Fig. 4 and Supplementary Material, Fig. S5). All reactions were performed in technical duplicate with a minimum of three biological replicates. Data are presented as mean ± sem in figures.

**Table:** 

Primer name	Primer sequence (5′-3′)
*MECP2* forward	TATTTGATCAATCCCCAGGG
*MECP2* reverse	CTCCCTCTCCCAGTTACCGT
*SOX2* forward	TACAGCATGTCCTACTCGCAG
*SOX2* reverse	GAGGAAGAGGTAACCACAGGG
*NANOG* forward	TGCAAGAACTCTCCAACATCC
*NANOG* reverse	CCTGGTGGTAGGAAGAGTAAAG
*HPRT* forward	GACCAGTCAACAGGGGACAT
*HPRT* reverse	CCTGAACCAAGGAAAGCAAAG
*GAPDH* forward	GGAGCGAGATCCCTCCAAAAT
*GAPDH* reverse	GGCTGTTGTCATACTTCTCATGG
*PPIA* forward	CTGAGCACTGGAGAGAAAGGA
*PPIA* reverse	TGTGAAGTCACCACCCTGAC

### RNA sequencing and data processing

RNA was isolated as described above and sent to Genewiz for RNA integrity assessment, library preparation, and sequencing on the Illumina HiSeq platform. For each sample, approximately 30 million 150 bp pair-end reads were generated. Raw reads were trimmed before mapping by Trimmomatic v0.39 using the adapter reference TruSeq3-PE.fa:2:30:10 [71]. Trimmed reads were aligned to GRCh38.p12 version 28 human genome assembly from GENCODE using STAR v2.7.9.a using all default parameters except –sjdbOverhang149. One sequenced sample was removed from further analysis due to poor RNA integrity score and alignment characteristics (Proband #3—no ASO, naïve treatment).

Read counts were then normalized and analyzed for differential gene expression using the DESeq2 package v1.34.0. Two initial baseline comparisons were made, each normalized independently. They contrast compare the naïve and scramble ASO-treated iNeurons per genotype (Supplementary Material, Fig. S3A and B). This acts as a negative control for any expression modulated by the scramble ASO compared to vehicle treatment. Then, a contrast comparing differences between unaffected control and MDS iNeurons (Fig. 3) establishes a disease signature. Both naïve and scramble control ASO-treated unaffected samples per cell line were used as control. This disease signature contrast was also performed using LimmaVoom with default parameters. The results for this comparison are in Supplementary Material, Table S1 [55]. Finally, eight contrasts were performed to examine the effects of *MECP2* ASO treatment. A design of ~(Genotype x Treatment x ASO dose) + Cell line was used to account for baseline cell line differences in gene expression. These eight contrasts are normalized per genotype and used the respective scramble ASO-treated samples as control. For each ASO and each dose per genotype, a separate contrast was calculated to generate a list of differentially expressed genes per condition. Normalization was done within either each genotype or contrast in consideration of the large sample size across the entire dataset and the human baseline genetic variance in expression. In total, five normalizations were performed: two to compare scramble ASO to naïve treatment (one per genotype), one to identify disease signature (MDS versus unaffected control), and two to compare scramble ASO to *MECP2* ASO treatments (one per genotype). In total, eleven contrasts were performed: two to compare scramble ASO to naïve treatment (one per genotype), one to identify disease signature (MDS versus unaffected control), and eight to compare scramble ASO to *MECP2* ASO treatments (one per genotype per ASO sequence and dose, Supplementary Material, Tables S2 and S3).

To assess reciprocal gene dysregulation in RTT neurons, we extracted the iNeuron data from a published dataset (https://zenodo.org/record/1226607#.YyFCwC-B0lV) (GSE107399) [37, 72]. These samples were merged based on gene symbol, and retained if the gene was expressed in both. Genes were considered a commonly differentially expressed gene if the *P-*adjusted value was less that 0.05.

To assess gene expression rescue upon ASO treatment, we first considered the genes dysregulated at baseline between unaffected controls and MDS iNeurons (Fig. 3). We computed the principal components on all the samples using only the disease signature genes identified by DESeq2. Next, we calculated the differences between the value of each sample’s principal component two to its matched average untreated sample. We averaged these differences per ASO across dosages per genotype to assess if global gene expression patterns were normalized in the direction towards the unaffected controls. We tested differences in principal component two using Student’s t-test between the normalized difference of the unaffected control samples to the ASO-treated samples (Supplementary Material, Fig. S6A). Next, we extracted the genotype-pooled normalized expression for each ASO sequence averaging the 6.6 μM and 20 μM dose samples for this signature of genes and merged these expression values with the genotype-pooled normalized expression for the unaffected control and MDS iNeurons as baseline. We then ordered expression values from 1 through 4 and clustered these rank-ordered patterns. Gene programs were considered rescued if the rank order correlated with *MECP2* dosage. To assess global rescue of the baseline genes dysregulated in MDS iNeurons, we calculated the log counts per million of the genes within the disease signature (Fig. 5). Next, we pooled these normalized counts per genotype and ASO treatment condition. The baseline dysregulation was calculated as the ratio of the normalized MDS counts per million to the normalized unaffected control counts per million. This calculation was then repeated for each ASO sequence and dosage by taking the ratio of the ASO-treated normalized counts per million to the control-treated counts per million for each genotype. A gene was considered partially rescued if the sign of the ratio was flipped (i.e. baseline ratio < 0 to an ASO-induced ratio > 0 and vice versa).

Heatmaps were generated using the pheatmap v1.0.12 package in R. Gene ontology analysis was performed by inputting the gene set of interest into the GSEA portal (www.gsea-msigdb.org). The top 10 significant (*p_adj_* < 0.05) gene ontology biological process terms were extracted per query and the *P*-adjusted value and percentage of gene list included per term were plotted.

### Transcription factor motif enrichment using HOMER

The gene list from the MDS gene signature (Fig. 3A) or gene list inversely dysregulated between RTT and MDS neurons (Fig. 3C) were input into the HOMER (v4.11.1) motif enrichment algorithm using the findMotifs.pl function with the human promoter set, considering a scanning start site of −1000 bp and end site of 100 bp, and motif lengths of 8, 10, and 12 nucleotides [57]. Motifs that had a *P*-value less than 1 × 10^−11^ were considered significantly enriched above background per the false positive flag from HOMER.

### Protein extraction and western blot

Protein lysates from cultured cells were collected by first washing culture in an appropriate volume of ice-cold PBS. PBS was aspirated and replaced with an appropriate volume of ice-cold RIPA lysis buffer (50 mM Tris–HCl pH 7.5, 150 mM NaCl, 1% Triton X-100, 0.5% sodium deoxycholate, 0.1% SDS, 5 mM EDTA, 1× Xpert phosphatase inhibitor cocktail [GenDEPOT #P32000], 1× Xpert protease inhibitor cocktail [GenDEPOT #P3100], and 1:1000 dilution of Peirce Universal Nuclease for Cell Lysis [ThermoFisher #88700]). Cell culture wells were mechanically lysed with a cell scraper, and all contents were transferred to a microcentrifuge tube. Samples were rotated for 20 min at 4°C. Lysates were centrifuged at maximum speed for 20 min at 4°C, and supernatants were transferred to a new microcentrifuge tube. Protein concentration was assessed using the Peirce BCA Protein Assay (ThermoFisher #23225) according to the manufacturer’s protocol. 15 μg of total protein was mixed with 1× NuPAGE LDS sample buffer (ThermoFisher #NP0007) with 1× NuPAGE Sample Reducing Agent (ThermoFisher #NP0004) and boiled at 95°C for 5 min. Prepared samples were run on a NuPAGE 4%–12% Bis-Tris gradient gel with MES SDS running buffer (ThermoFisher #NP000202). Separated proteins were transferred to a 0.45 μm pore-size Immobilon-FL PVDF membrane (MilliporeSigma #IPFL0010) using NuPAGE transfer buffer (ThermoFisher #NP0061) with 10% methanol at wet-transfer electrophoretic transfer conditions of 100 V constant for 2 h at 4°C. The membranes were blocked in 0.5× Odyssey Blocking Buffer (TBS; LiCOR #927-50000) for 1 h at room temperature. Membranes were then probed with primary antibody diluted in 0.5x blocking buffer +0.1% Tween-20. The following antibodies and dilutions were used: anti-MeCP2 (rabbit monoclonal D4F3, Cell Signaling Technology #3456, RRID:AB_2143849; 1:1000 dilution), anti-Vinculin (mouse monoclonal hVIN-1, MilliporeSigma #V9131, RRID: AB_477629; 1:20000 dilution), anti-phospho-S6 ribosomal protein (Ser235/236) (rabbit monoclonal D57.2.2E, Cell Signaling Technology #4858, RRID: AB_916156, 1:1000 dilution), anti-total S6 ribosomal protein (mouse monoclonal 54D2, Cell Signaling Technology #2317, RRID: AB_2238583, 1:1000 dilution), anti-total mTOR (rabbit monoclonal 7C10, Cell Signaling Technology #2983, RRID: AB_2105622, 1:1000 dilution), anti-phospho-4E-BP1 (Thr37/46) (rabbit monoclonal 236B4, Cell Signaling Technology #2855, RRID: AB_560835, 1:1000 dilution), anti-GAPDH (mouse monoclonal 6C5, Advanced Immunochemical #2-RGM2, RRID: AB_2721282, 1:20000 dilution), anti-pan AKT (rabbit monoclonal C67E7, Cell Signaling Technology #4691, RRID: AB_915783, 1:1000 dilution), anti-α tubulin (chicken polyclonal, abcam #ab89984, RRID: AB_10672046, 1:20000 dilution), anti-phospho-c-Raf (Ser259) (rabbit polyclonal, Cell Signaling Technology #9421, RRID: AB330759, 1:1000 dilution), anti-phospho-GSK3β (Ser9) (rabbit monoclonal D85E12, Cell Signaling Technology #5558, RRID: AB_10013750, 1:1000 dilution), and anti-phospho-CREB (Ser133) (rabbit monoclonal 87G3, Cell Signaling Technology #9198, RRID: AB_2561044, 1:1000 dilution). Membranes were washed four times with TBS-T and incubated in LiCOR secondary antibodies (1:20000 dilution) in 0.5× blocking buffer +0.1% Tween-20 + 0.1% SDS for 1 h at room temperature. Membranes were washed four times with TBS-T and one time with TBS. Membranes were then imaged on a LiCOR CLx imager. Band intensities were quantified using the Analyze Gel commands in Fiji. MeCP2 intensities were normalized to vinculin intensities. Data are presented as mean ± sem in figures.

## Supplementary Material

Bajikar_etal_HMG_revision_supplemental_final_v3_ddae135

SupplementalTable1_ddae135

SupplementalTable2_ddae135

SupplementalTable3_ddae135

SupplementalTable4_ddae135

SupplementalTable5_ddae135

## Data Availability

Raw RNA-sequencing data cannot be made publicly available due to privacy and consent concerns. Raw aligned counts and normalized processed expression are deposited in the Zenodo repository (https://zenodo.org/records/11637510; 10.5281/zenodo.11637510). The RTT iNeuron RNA-sequencing data is available through the Gene Expression Omnibus (GSE107399) and processed data through: https://zenodo.org/record/1226607#.YyFCwC-B0lV. This study does not report any original code and the utilized code to generate the analyses in this manuscript is available from the lead contact upon request. Any additional information required to reanalyze the data reported in this manuscript is available from the lead contact upon request.

## Notes

*Conflict of interest statement:* FR and PJN are paid employees of Ionis Pharmaceuticals. HYZ is on the board of directors of Regeneron Pharmaceuticals, a scientific advisor to the Column Group, Cajal Neuroscience and to BridgeBio and receives funds for research not included in this study from UCB Pharmaceuticals and Ionis Pharmaceuticals. HYZ is a co-founder of Cajal Neuroscience. HYZ and YS are inventors on patent/patent application (US 16/535888) held/submitted by the Baylor College of Medicine that covers ‘Compositions and methods for modulating *MECP2* expression’. The authors declare that they have no other competing interests.

## Statistical analysis

Statistical analyses were conducted in GraphPad Prism for biochemical data or R for RNA-sequencing data. The number of biologically different cell lines (*n*) and specific statistical tests for each experiment are indicated in the figure legends. Student’s t-test was used for single genotype comparisons. Two-way ANOVA was used to assess genotype, time, and ASO dosage effects in biochemical measurements.
