# Supplementary material for: Modeling antisense oligonucleotide therapy in MECP2 duplication syndrome human iPSC-derived neurons reveals gene expression programs responsive to MeCP2 levels
Source: Hum Mol Genet. 2024 Sep 15;33(22):1986–2001. doi: 10.1093/hmg/ddae135 (PMC11555823; doi:10.1093/hmg/ddae135)
Supplement: Bajikar_etal_HMG_revision_supplemental_final_v3_ddae135 [file bajikar_etal_hmg_revision_supplemental_final_v3_ddae135.pdf]

A

	Subject	Sex	ChrX dup. coordinates (hg19)	Age (yrs)	Publication
●	Proband 1	M	153,183,739 - 153,623,000	24	This manuscript
■	Proband 2	M	153,183,739 - 153,623,000	22	This manuscript
▲	Proband 3	M	152,834,323 - 155,251,054	7	This manuscript
◆	Proband 4	M	153,093,425 - 153,558,775	6	This manuscript
●	Unaffected 1	M	N/A	7	Gillentine et al.
■	Unaffected 2	M	N/A	20	This manuscript
▲	Unaffected 3	M	N/A	16	Kho et al.
◆	Unaffected 4	M	N/A	22	Kho et al.

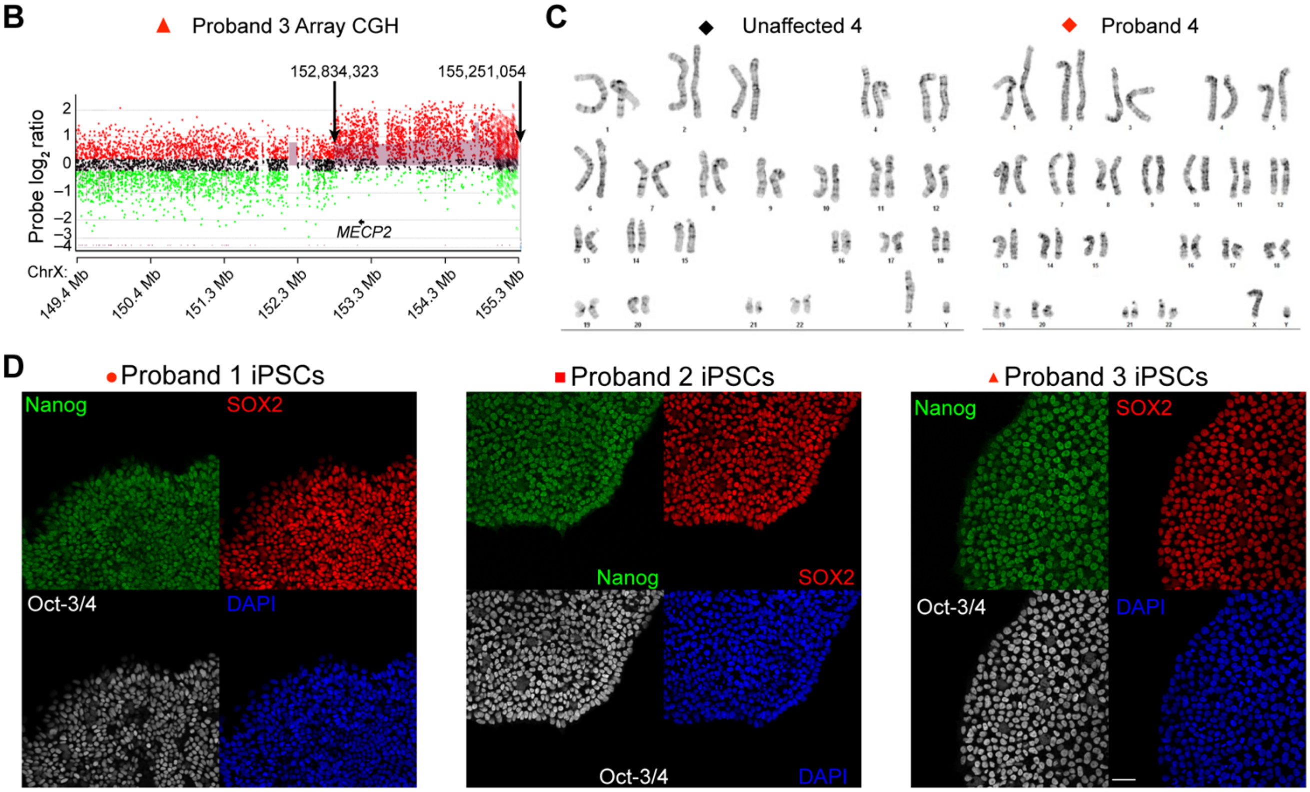

Figure S1. Characterization of patient-derived *MECP2* duplication syndrome induced pluripotent cells (iPSCs). (A) Summary table of the genetics and cell lines demographics from probands and unaffected controls in this study. Shape represents a given individual throughout the manuscript. (B) Example array CGH quantification of genomic duplication on X-chromosome for Proband 3. (C) Karyotype is preserved after reprogramming. Representative karyotype of one unaffected and one MDS patient derived iPSC line. (D) Validation of pluripotency marker expression (Nanog, SOX2, Oct-3/4) in MDS derived patient iPSCs. Scale bar = 100 µm.

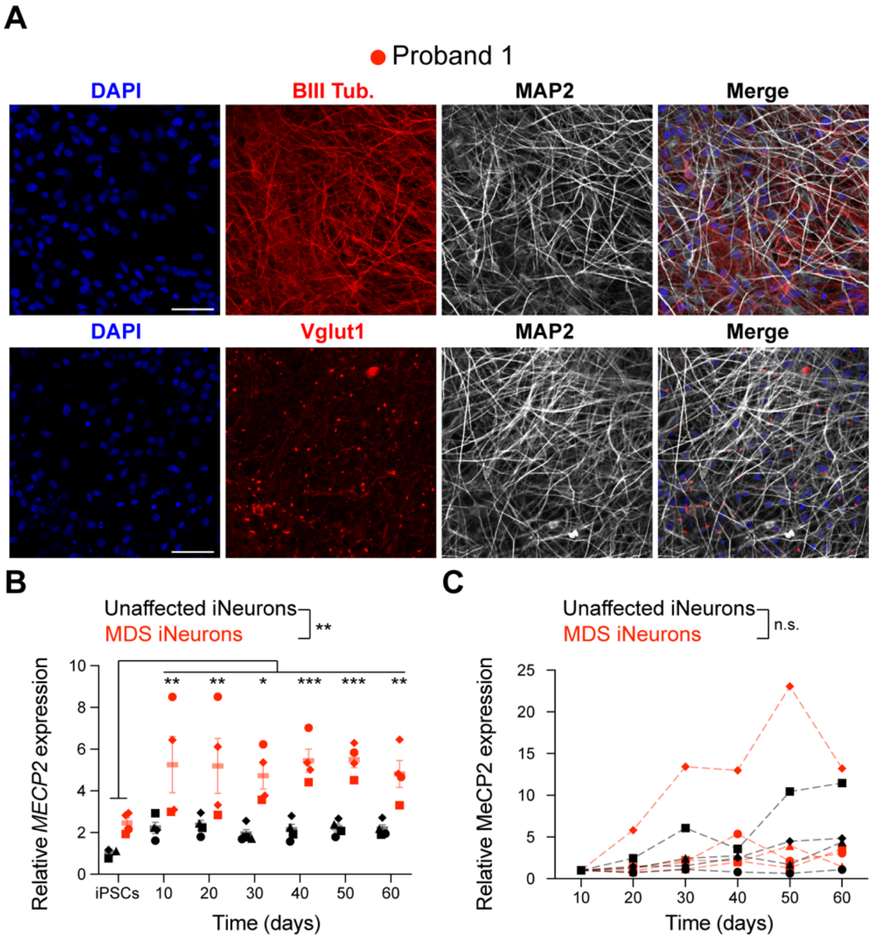

Figure S2. Characterization of *MECP2* duplication syndrome iNeurons derived using NGN2-directed differentiation. (A) Immunofluorescence of excitatory neuronal (Vglut1) and neuronal cytoskeletal (MAP2 and BIII tubulin) markers in MDS iNeurons. Images are from one patient line (Proband #1), representative of multiple differentiations across patient lines. Staining was performed after eight weeks in culture. Scale bar = 50 µm. (B) Relative *MECP2* expression normalized to iPSC expression per patient. Data from days 10-60 are the same as displayed in Figure 2B. Differences in RNA expression were assessed by two-way ANOVA and multiple comparisons test to iPSC expression (**$P_{genotype} < 0.01$) (C) Fold-change of MeCP2 protein expression normalized to D10 for each patient from the relative quantification displayed in Figure 2D.

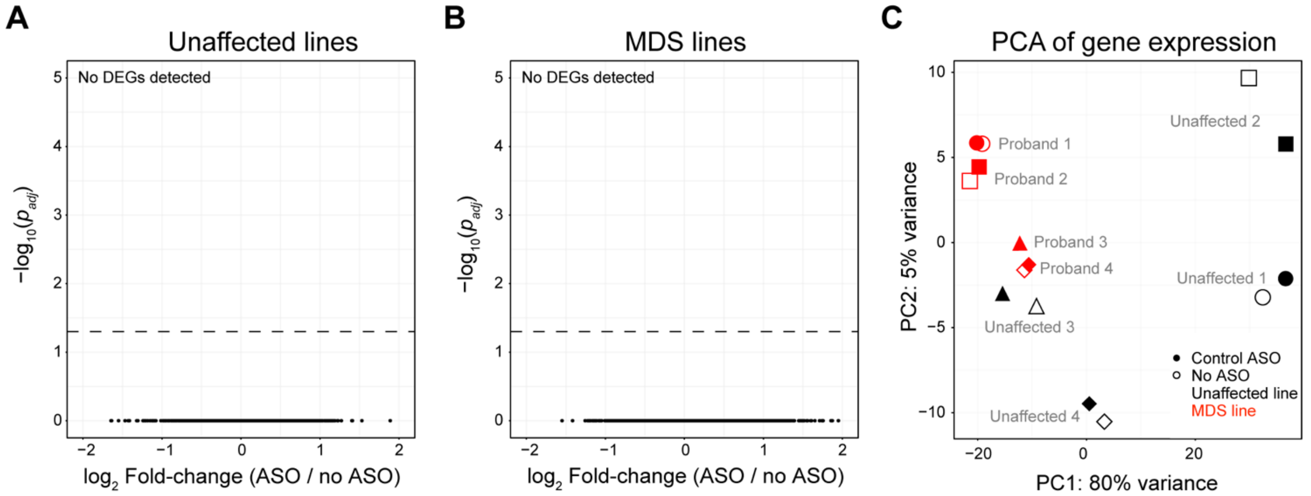

Figure S3. Variation analyses of gene expression changes in iNeurons. (A-B) Volcano plot denoting significant ($p_{adj} < 0.05$) gene expression changes between scramble treated and naïve iNeurons from either unaffected control (A) or MDS (B) genotypes (n = 3-4 biological replicates). Horizontal dashed line denotes p_{adj} = 0.05. (C) Principal component analysis (PCA) of gene expression patterns. Principal components (PC) were calculated on the differentially expressed genes and plotted in reduced space along PC#1 and #2. Samples are colored by genotype, the shape denotes proband per symbols used in Figure 1 and Figure S1, and scramble ASO treatment or naïve culture is denoted by filled or open shape, respectively.

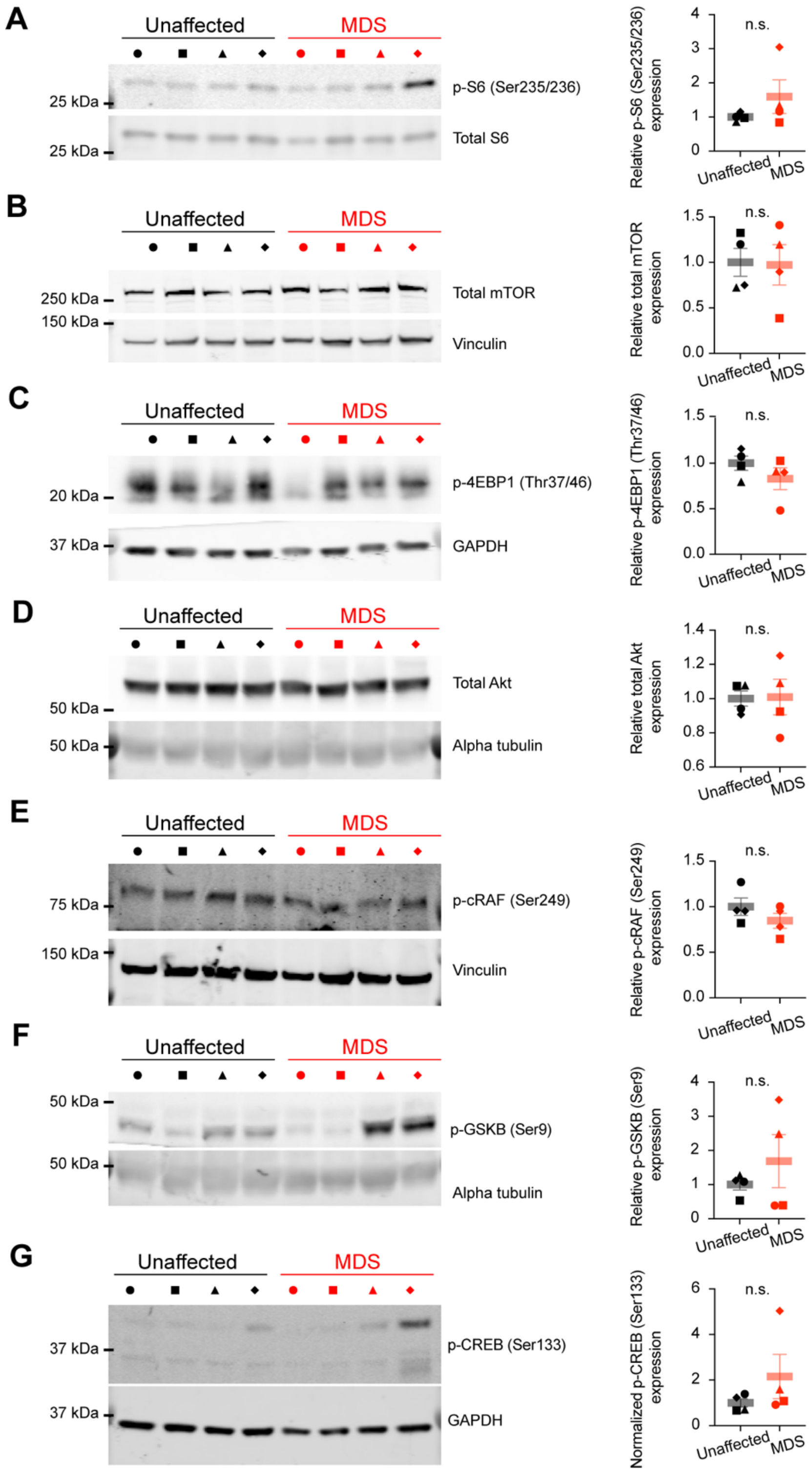

Figure S4. The levels of components of the AKT/mTOR and CREB pathways are not significantly altered between MDS and unaffected control iNeurons. Western Blot analysis of unaffected and MDS iNeuron lysates from neurons cultured for 40 days were profiled for the indicated marks (see Methods). Target protein signal (top blot) was normalized to loading control (lower blot) per probe; the normalized integrated intensity was analyzed using Student's t-test by genotype. n.s. – not significant.

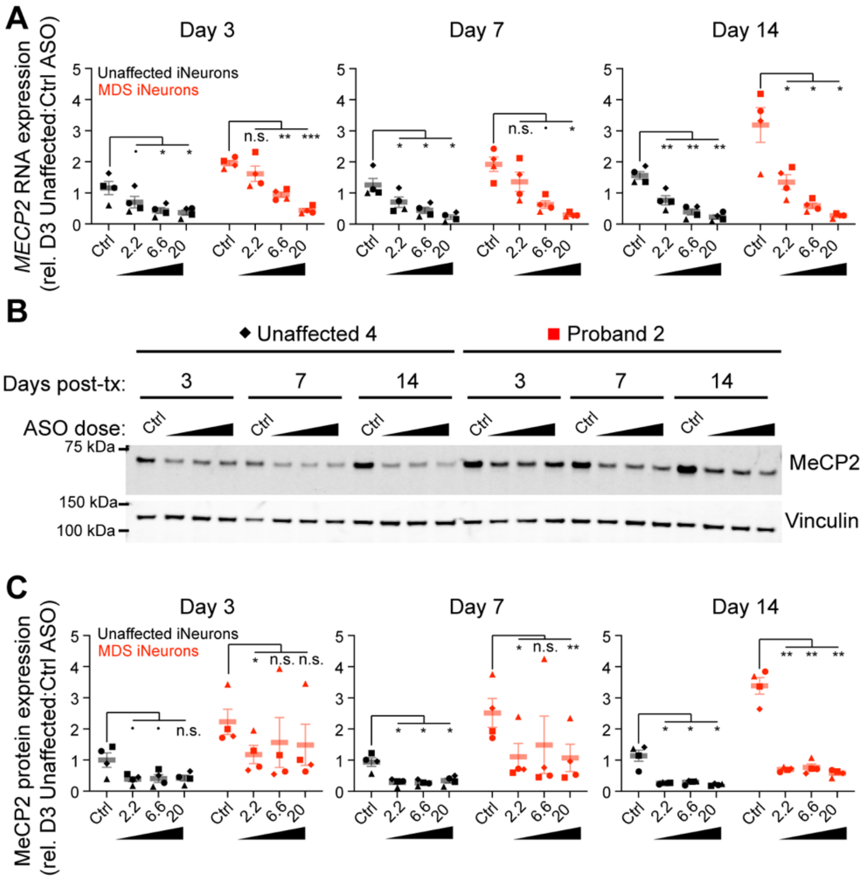

Figure S5. Antisense oligonucleotide treatment acutely reduces *MECP2* RNA expression and MeCP2 protein levels in culture using a second anti-*MECP2* ASO (ASO#2). ASO in three doses (2.2, 6.6, and 20 µM) was introduced into the media starting 30 days after culture and supplemented with fresh ASO at 3, 7, and 10 days after the first dose. Control scramble ASO was used at the maximum dose. RNA and protein lysates were collected 3, 7, and 14 days after first dose of ASO. (A) *MECP2* RNA expression after ASO treatment as measured by qRT-PCR. (B-C) MeCP2 protein expression after ASO treatment as measured by Western blot. (B) Representative MeCP2 protein expression during ASO treatment for one unaffected (black) and MDS (red) as measured by Western blot. Vinculin was used as a loading control. (C) Quantification of MeCP2 signal intensity normalized to vinculin signal intensity per genotype per individual. Graphs display mean ± sem of $n = 4$ biological replicates per genotype, individuals are depicted with a separate shape. Data are normalized to RNA or protein quantification of the unaffected neurons treated with scramble control ASO at day three of treatment. Data were analyzed by two-way ANOVA and Sidak's multiple comparisons test to scramble controls (· $P < 0.1$, * $P < 0.05$, ** $P < 0.01$),

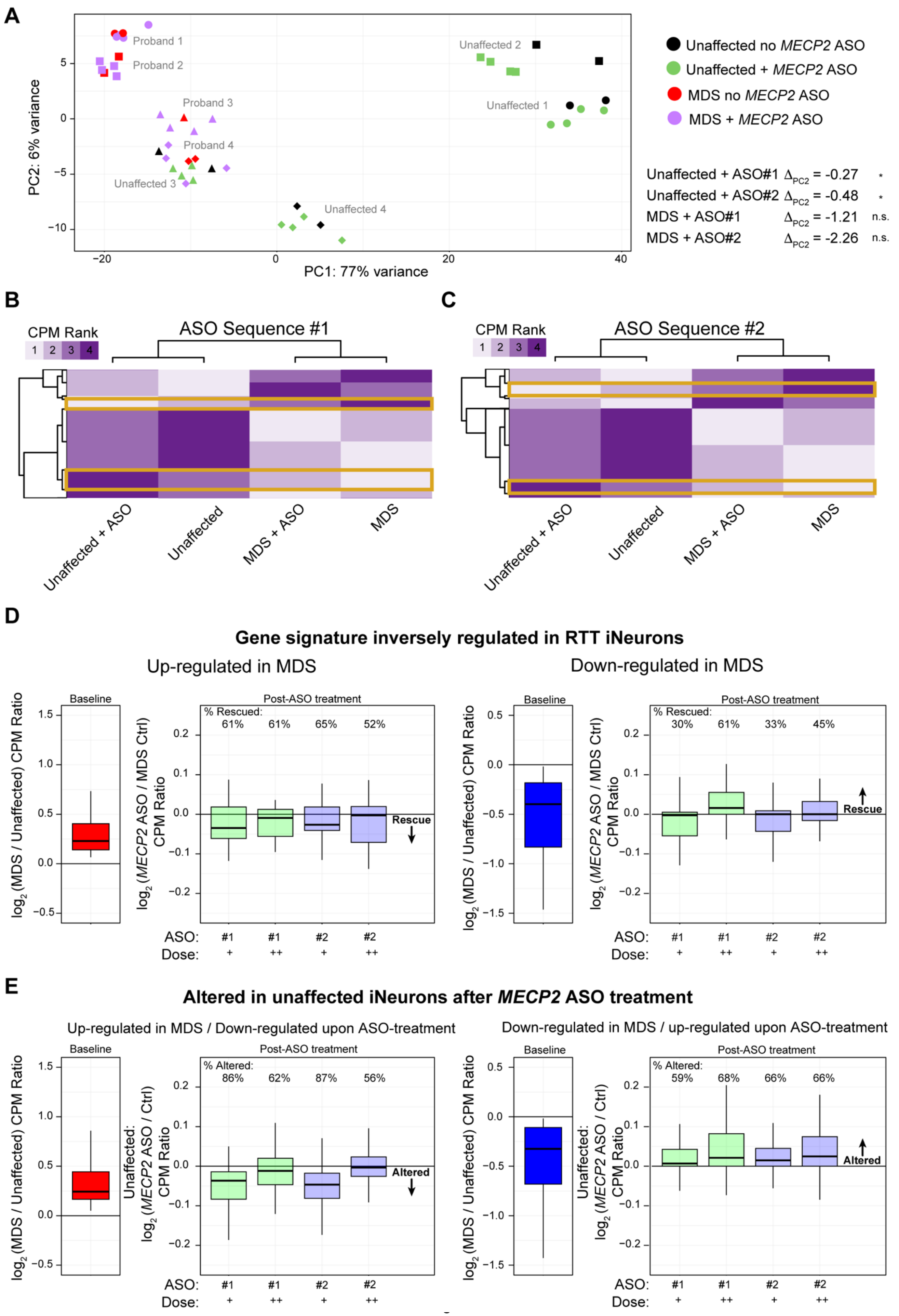
A
Proband 1
Proband 2
Proband 3
Proband 4
Unaffected 1
Unaffected 2
Unaffected 3
Unaffected 4
PC1: 77% variance
PC2: 6% variance
Unaffected no MECP2 ASO
Unaffected + MECP2 ASO
MDS no MECP2 ASO
MDS + MECP2 ASO
Unaffected + ASO#1 Δ_{PC2} = -0.27 *
Unaffected + ASO#2 Δ_{PC2} = -0.48 *
MDS + ASO#1 Δ_{PC2} = -1.21 n.s.
MDS + ASO#2 Δ_{PC2} = -2.26 n.s.
B
CPM Rank
ASO Sequence #1
Unaffected + ASO
Unaffected
MDS + ASO
MDS
C
CPM Rank
ASO Sequence #2
D
Gene signature inversely regulated in RTT iNeurons
Up-regulated in MDS
Down-regulated in MDS
Baseline
Post-ASO treatment
% Rescued:
61% 61% 65% 52%
30% 61% 33% 45%
Rescue
log_2 (MDS / Unaffected) CPM Ratio
log_2 (MECP2 ASO / MDS Ctrl) CPM Ratio
ASO: #1 #1 #2 #2
Dose: + ++ + ++
E
Altered in unaffected iNeurons after MECP2 ASO treatment
Up-regulated in MDS / Down-regulated upon ASO-treatment
Down-regulated in MDS / up-regulated upon ASO-treatment
% Altered:
86% 62% 87% 56%
59% 68% 66% 66%
Altered
Unaffected: log_2 (MECP2 ASO / Ctrl) CPM Ratio

Figure S6. Comparative analysis of ASO-mediated gene expression changes across genotypes and identification of programs of genes responsive to MeCP2 dosage. (A) Principal component analysis of MDS disease signature across ASO treated samples. The change in principal component two for each ASO sequence aggregated by dosage and genotype is shown to the right. Differences in principal component 2 were assessed using Student's t-test within group. (*) $P < 0.05$, n.s. – not significant (B-C) Clustering ranked order of average counts per million per transcript after ASO treatment per gene dysregulated at baseline in MDS. CPMs were ordered per row 1-4 and clustered by row and column. Yellow boxes highlight gene expression programs that show monotonic increase or decrease between Unaffected+ASO, Unaffected, MDS+ASO, and MDS gene expression. Left clustergram shows results from ASO sequence #1 and right clustergram shows results from ASO sequence #2. (D) Alteration of the subset of MDS disease signature genes that are shared with RTT iNeurons. The boxplots depict the distribution of the $\log_2$ ratio of the average counts per million between either MDS and unaffected neurons (left inset panels) or anti-*MECP2* ASO treated and MDS control (scramble ASO and naïve) neurons. Genes with a ratio above 0 are upregulated in the numerator, while below 0 are downregulated in the numerator (right inset panels). Genes that were up-regulated in MDS lines at baseline and that were qualitatively reduced after ASO treatment fall below 0 on the left inset panel. Genes that were down-regulated in MDS lines at baseline and that were qualitatively increased after ASO treatment fall above 0 on the right inset panel. These directions, highlighted by the black arrows, represent expression levels trending towards the unaffected control expression levels. The percent of genes trending towards rescue, as depicted by the direction of the black arrow, for either up- or down-regulation are shown above each ASO treatment condition. (E) Genes that are altered in ASO-treated unaffected control lines and that are opposite to the MDS disease signature. The boxplots depict the distribution of the $\log_2$ ratio of the average counts per million between either MDS and unaffected neurons (left inset panels) or unaffected neurons treated with either anti-*MECP2* ASO or control (scramble ASO and naïve) neurons. Genes with a ratio above 0 are upregulated in the numerator, while below 0 are downregulated in the numerator (right inset panels). Genes that were up-regulated in MDS lines at baseline and that were qualitatively reduced after ASO treatment in unaffected control lines fall below 0 on the left inset panel. Genes that were down-regulated in MDS lines at baseline and that were qualitatively increased after ASO treatment in unaffected control lines fall above 0 on the right inset panel. The black arrows highlight the direction of altered gene expression change in the unaffected control lines matching the direction of the anti-*MECP2* ASO treated MDS lines. These sets represent genes that correlate with *MECP2* expression independent of genotype.

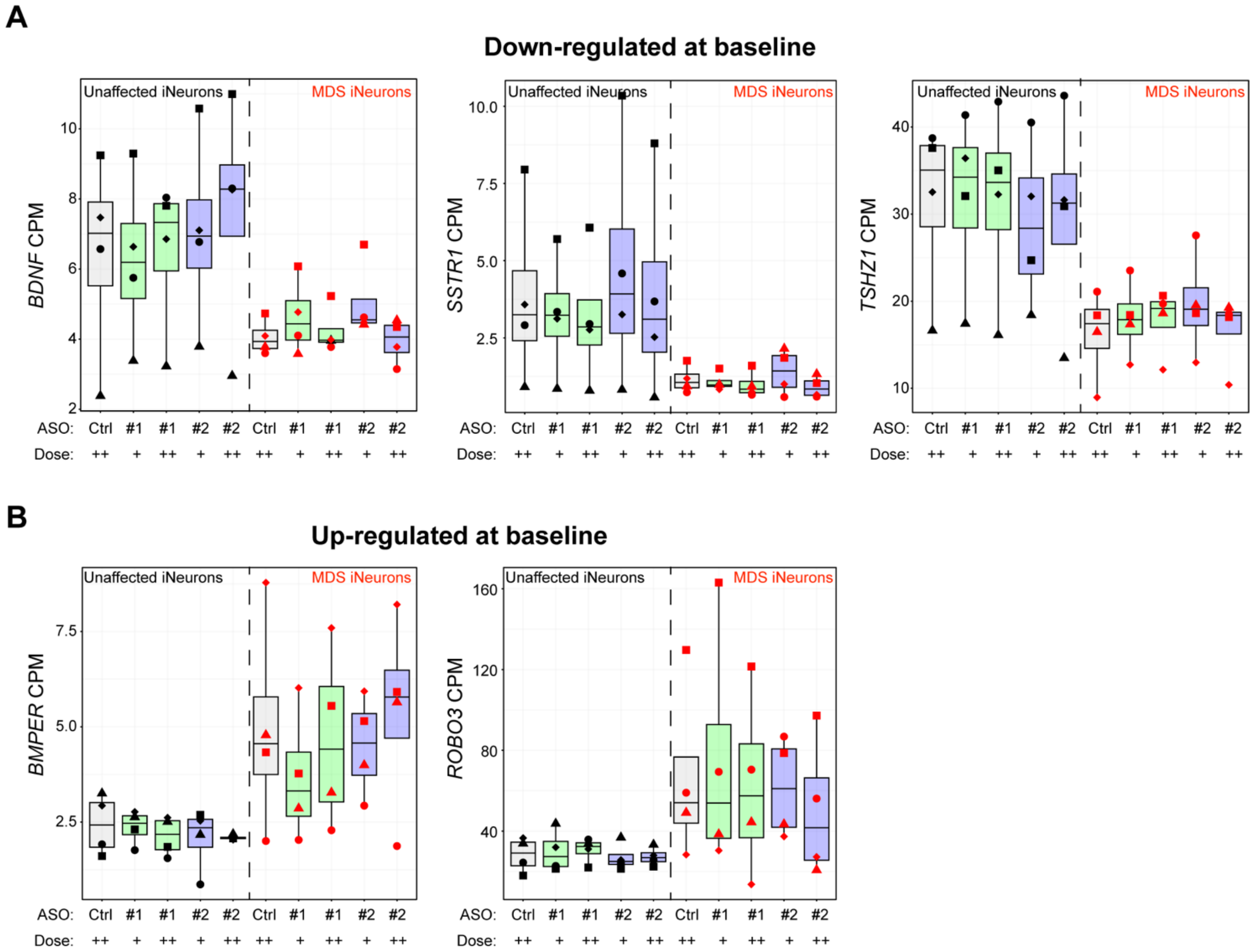

Figure S7. Examples of genes putatively regulated by MeCP2 but unchanged upon two weeks of anti-*MECP2* ASO treatment in iNeurons. Counts per million (CPM) of *BDNF* and a sample of top *MECP2* gene targets from MECP2pedia (www.mecp2pedia.org) were not found to be altered after ASO treatment in either genotype. (A) Example target genes that are unaltered after ASO treatment that are down-regulated in MDS iNeurons at baseline. (B) Example target genes that are unaltered after ASO treatment that are up-regulated in MDS iNeurons at baseline. Values for CPM in unaffected (black) and MDS (red) lines for control scramble ASO (Ctrl) or each anti-*MECP2* sequence across two doses (+ is 6.6 μM dose, ++ is 20 μM dose, control scramble ASO used at 20 μM dose). Graphs display boxplots of n = 4 biological replicates per condition, individuals are depicted with a separate shape.
